# Influence of the Mesoporosity of Silica Carrier Materials on the Performance of an Immobilized Organocatalyst in Heterogeneous Catalysis

**DOI:** 10.1021/acsami.4c19398

**Published:** 2025-04-10

**Authors:** Aline Trommer, Janis Hessling, Peter R. Schreiner, Monika Schönhoff, Bernd M. Smarsly

**Affiliations:** † Institute of Physical Chemistry, Justus Liebig University, D-35392 Giessen, Germany; ‡ Institute of Physical Chemistry and Center of Soft Nanoscience, Westfälische Wilhelms- University, D-48148 Münster, Germany; § Institute of Organic Chemistry, Justus Liebig University, D-35392 Giessen, Germany; ∥ Center for Materials Research, Justus Liebig University, D-35392 Giessen, Germany

**Keywords:** heterogeneous organocatalysis, diffusion, mass transport assessment, physisorption, porous silica, pulse-field gradient NMR, Weisz−Prater-Criterion

## Abstract

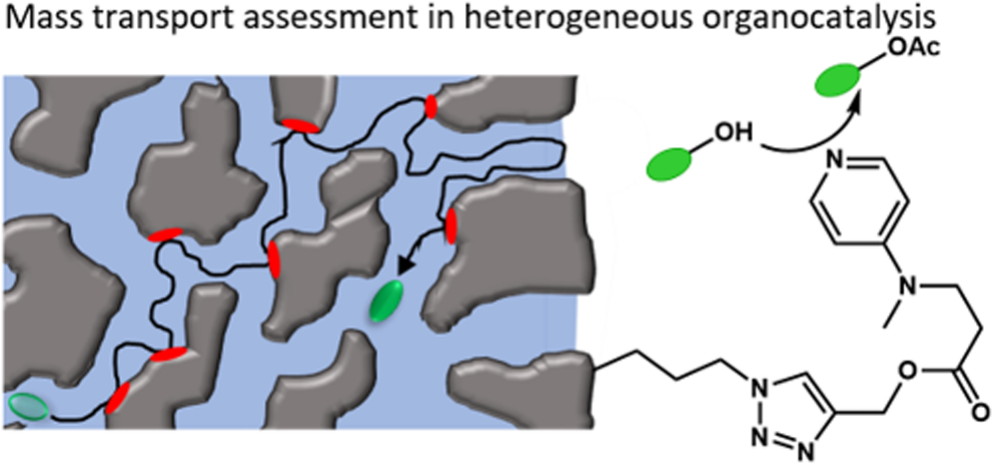

While the immobilization of organocatalysts in mesoporous scaffolds brings significant benefits, the relationship between mesopore diameter and the performance of the catalyst material is still a matter of research. Here, we studied the esterification of α-tocopherol (**TP**) and 1-phenylethanol (**PE**) using a versatile and well-known organocatalyst, 4-(dimethylamino)pyridine-(DMAP), being immobilized in mesoporous spherical silica particles, which possess three different mesopore diameters (6, 10, and 30 nm), but an identical particle diameter (5 μm). In order to analyze the interplay of transport and reactivity, the mesoporosity was thoroughly studied by advanced physisorption analysis, especially desorption hysteresis scanning. Furthermore, we used pulsed-field gradient (PFG) NMR for the determination of the intraparticular self-diffusion behavior of the chosen alcohols as well as their acetalyzed forms and also to identify their interaction with the polar silica surface. The catalytic performance was tested in packed-bed columns (continuous flow) as well as under batch conditions. Based on catalytic parameters and experimental diffusion coefficients, the Weisz–Prater-Criterion parameter Φ_WP_ was calculated for identifying potential mass-transport limitations for each material, to evaluate the influence of the mesopore diameter on the catalytic properties. In conclusion, we demonstrate that the observed poor catalytic performance in the esterification reaction of α-tocopherol, in the case of the material with the smallest average mesopore size (6 nm), is due to hindered mass transport. The combination of physisorption and diffusion analysis suggests that this limitation is on the one hand caused by bottleneck-like connecting pores and on the other hand by liquid–surface interactions. However, quite high product yields are observed for the 10 nm- and 30 nm-mesoporous particles under flow conditions, which correlates well with the self-diffusion coefficients obtained from PFG NMR, showcasing the feasibility of immobilized organocatalysts in organic synthesis.

## Introduction

The field of organocatalysis has attained significant relevance because of various benefits in terms of product selectivity and activity as well as the potential for sustainable synthesis, as also evident from the Nobel Prizes in 2021 devoted to asymmetric organocatalysis.^1^ Compared to homogeneous organocatalysis, the immobilization of organocatalysts on solid support makes product workup and recycling easier, potentially preventing the contamination of product with catalyst or reactant residues. Also, porous materials equipped with immobilized organocatalysts allow for repeated usage. Hence, organocatalysis using organocatalysts immobilized on high-surface area materials, in particular, in continuous flow setups, shows high promise in terms of ecological and economic considerations.^2,3^

Mesoporous silica is a suitable carrier material for immobilized catalysts because of its stability, inertness to most reaction conditions in organocatalysis, and large surface area, which allows for high catalyst loadings. Therefore, it has been used as a carrier material in various morphologies.^4−12^ The use of catalyst-functionalized mesoporous materials in a continuous flow setup brings additional advantages: optimized flow-properties of a reactor coupled with a large surface-to-volume ratio lead to short reaction times, and separate product purification and catalyst recycling steps can be avoided since the catalyst material stays inside the reactor. By combining various functionalized materials, a continuous multistep reaction is possible, and by numbering-up or scaling-out, the process can be easily scaled up.^13−15^ Yet, for applications in continuous flow setups, the carrier materials have to meet additional requirements. For example, the material must not exhibit high pressures at high flow rates and, at the same time, should enable a quick flow-through of reactant and product molecules.

4-(Dimethylamino)pyridine (DMAP) is a nucleophilic catalyst used for various reactions, for example, the acylation of alcohols and amines.^16−18^ Hence, because of this versatility, DMAP serves as a suitable model catalyst for obtaining fundamental insight into the relationship between the mesopore space and catalytic performance. Beyond this academic aspect, DMAP is of significant relevance in regards to application, as relevant large-scale organic reactions use DMAP as a catalyst.^19,20^ We recently showed that DMAP-functionalized mesoporous silica particles and monoliths, both specifically optimized and commercialized for HPLC, show excellent performance and stability in continuous flow catalysis, as exemplified for the esterification of 1-phenylethanol (**PE**).^21^

Yet, in previous work, only one mesopore diameter was studied for both types of materials used, i.e., silica particles and monoliths (mesopore size ca. 10 and 12 nm, respectively). However, to expand the substrate scope in the direction of using larger reactant molecules and materials with higher surface area and—concomitantly—smaller mesopore diameters, fundamental insight into the relationship between the mesopore size and potential connectivity restrictions and transport limitations is needed. In particular, targeting the use of commercially available silica materials would benefit from identifying an optimum mesopore dimension with respect to transport and the corresponding catalytic performance.

In liquid chromatography, it is known that diffusion into and within the mesopores is restricted for pore diameters smaller than approximately ten times the molecules’ dimensions, which significantly decreases the separation performance of HPLC columns.^22^ Accordingly, the yield of reactions performed using immobilized organocatalysts might suffer from too small mesopore diameters or too small connecting necks. Yet, there are only few studies varying the mesopore size in materials equipped with immobilized organocatalyst used in flow.^22−26^

To evaluate the transport of molecules in porous materials, the tortuosity τ is a commonly employed concept.^27−29^ It describes the geometric restriction that the pore structure imposes on the transport of a molecule in a porous material. It can be defined as the ratio of the effective path length (*L*_eff_) in the porous material over the direct path length (*L*_0_) according to τ = *L*_eff_/*L*_0_. Due to the enhancement of the path length, the geometric restriction therefore leads to an apparent diffusion coefficient (*D*_eff_) of the molecules, which is lower than the diffusion coefficient of that molecule in bulk (*D*_0_). This can be described by using the relation of the mean-square displacement with the diffusion coefficient, according to τ = (*D*_0_/*D*_eff_)^1/2^. We note here that an alternative definition of tortuosity is equally often used in literature, where it is given as the ratio of diffusion coefficients, τ = *D*_0_/*D*_eff_.^29^ While the concept of tortuosity describes the geometrical hindrance in a porous material, it often fails to accurately describe transport in porous materials because it does not consider interactions of the diffusing molecules with the pore wall.^30,31^ The diffusion of molecules bearing functional groups interacting with the material may be additionally hindered by surface–liquid interactions. In this case, the diffusion coefficient (*D*_eff_) is smaller than predicted by the tortuosity parameter. Pulsed field gradient (PFG) NMR is a powerful tool to determine the diffusion coefficients of molecules confined in the porous material and in bulk.^32−34^ Hence, applying a suitable experimental procedure combined with models for data fitting and interpretation, we are able to accurately describe the intraparticle transport of the different molecules in our silica material.

The transport properties of porous materials are critical for designing efficient fixed-bed reactors for the application in separation chromatography and heterogeneous flow catalysis.^35,36^ In the case of the used packed-bed reactor, the mass transport is accomplished in the interparticle void, whereas the actual catalysis occurs in the mesoporous space, i.e., within the spherical particles.^37^ If the transport of molecules into and within the mesopore space is hindered and limited, then the porosity of the carrier materials can have a huge impact on the rate of the catalyzed reaction. A frequently applied approach to identify and evaluate intraparticle diffusion limitation effects is the calculation of the so-called Weisz–Prater-Criterion Φ_WP_.^38^ By determining the ratio of the observed reaction rate to the rate of effective diffusion of the reagents inside the pores, an assessment of the internal transport limitations is possible.^39−41^ Evidently, this concept requires accurate values for the intraparticular self-diffusion coefficient, which often can only be estimated.

**Figure 1:**
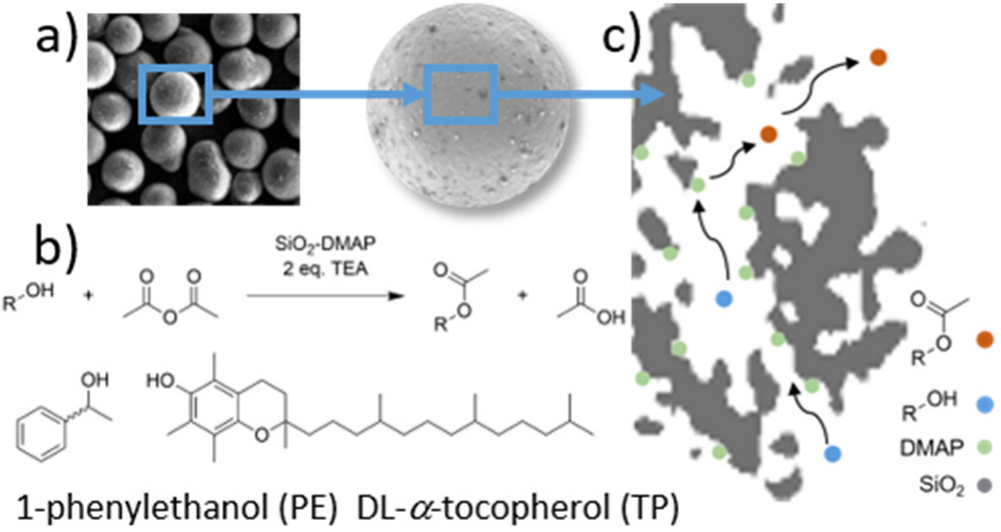
**a** SEM images with different magnifications showing the spherical silica carrier material. **b** Esterification of 1-phenylethanol (**PE**) and α-tocopherol (**TP**) to respective acetates by DMAP immobilized on porous silica particles. Triethylamine is added as an auxiliary base. **c** Scheme highlighting the diffusion of molecules in the mesopores of the functionalized silica particles.

In order to study the interplay of mesopore size and dimension of the reactant molecules in their impact on catalytic performance, we chose the low-molecular-weight alcohol 1-phenylethanol (**PE**) and the much larger molecule α-tocopherol (**TP**), a natural Vitamin E component containing a comparably long alkyl chain. A visual representation of the overall reaction scheme is shown in Figure 1b. Not only does **TP** provide a suitable example for a sterically demanding reactant, but the esterification of **TP** to **TP**-acetate is also an industrially relevant application, because the acetate group leads to a better shelf life, while being easily cleaved once ingested into, e.g., the human body.^42−44^Figure 1 displays SEM images of our chosen carrier materials, which are commercially available fully mesoporous LiChrospher and PerfectSil particles featuring a diameter of 5 μm and a uniform spherical particle shape that leads to dense and homogeneous packing in a column. Since previous studies on immobilized catalysts showed promising catalytic properties for a pore size of 10 nm,^21,45^ particles with average mesopore diameter of 6, 10, and 30 nm were used for the present study, while the average particle diameter (5 μm) is identical. Thus, a potential difference in the organocatalytic performance of these three materials allows us to unravel the impact of the mesopore dimensions and also their mutual connection in regard to diffusion limitations. In addition, we perform detailed diffusion studies by PFG NMR. Taking into account the special features and boundary conditions of porous spherical particles, we use a suitable model to extract the intraparticle diffusion coefficients for different substrate sizes and pore sizes. The diffusion of substrate molecules to/from the active sites on the mesopore surface might be hindered by the combination of a large dimension of the reactant and small connecting necks between pores, thus resulting in a poorer performance. Hence, our study targets fundamental insight into the impact of the mesopore dimension and connectivity and the resulting diffusive transport on catalyst performance, which might result in advances in the industrial implementation of heterogeneous organocatalysis.

## Materials and Methods

### Silica Functionalization

The synthesis of (3-azidopropyl)trimethoxysilane and the DMAP click-derivative was done as described in the literature. The reaction scheme as well as characterization data are depicted in the Supporting Information (Figures S1–S2).^21^

Initially, 1.5 g of the silica material (LiChrospher Si 60/100, PerfectSil300, 5 μm) was heated under reduced pressure to 100 °C for 2 h. Next, 12 mL of dry toluene, 10 w% triethylamine (900 μL, 663 μL, 7.2 mL) and (3-azidopropyl)trimethoxysilane (90 μL, 63 μL, 750 μL) were added, and the suspension was heated to 80 °C overnight in a shaking water bath. The particles were then washed with dichloromethane, methanol, and methanol/water (1:1) and dried.

1.0 g samples of azide-functionalized silica particles featuring three different mesopore diameters (6/10/30 nm) were suspended in 12 mL of dry toluene. Two tips of a spatula Bromotris(triphenylphosphine)copper(I), 258/245/360 mg (1.2/1.1/1.6 mmol, 2 eq. in relation to azide on silica) of the DMAP derivative, and 600 μL (3.4 mmol) N,N-diisopropylethylamine were added. The solution was heated to 50 °C in a shaking water bath for 3 days. The particles were washed with acetonitrile, methanol, Na_2_EDTA (5 wt %), and water and were then dried. The resulting DMAP loading was determined by elemental analysis (see ESI file for further information).

### Reaction Setup

**Batch conditions**: For heterogeneous catalytic experiments under batch conditions, the catalyst amount was calculated based on the elemental analysis, in particular, the nitrogen content. All reactions were carried out at room temperature. Aliquots were taken at different times (15/30/60/90/120/150 min). For each aliquot, the catalyst was separated from the reaction solution by centrifugation at 8000 rpm for 5 min.

**PE**: For catalytic experiments in batch, 72 μL (0.6 mmol) of 1-phenylethanol, 125 μL (0.9 mmol, 1.5 equiv) of triethylamine, and 85 μL (0.9 mmol, 1.5 equiv) of acetic acid anhydride were suspended in 1.72 mL of toluene, and 1 mol% (respective to the alcohol) of the 6/10/30 nm porous material, respectively, (23.2/23.2/33.3 mg) was added.

**TP**: For the batch catalysis of α-tocopherol, 5 mol% (respective to the alcohol) of each material (14.8/10.2/15.1 mg) was used. A reaction solution was prepared by suspending 25.8 mg (0.06 mmol) of α-tocopherol with 17 μL (0.12 mmol, 2 equiv) of triethylamine and 11 μL (0.12 mmol, 2 equiv) of acetic acid anhydride in 2.97 mL of toluene.

**Flow conditions**: For catalytic tests in flow, the functionalized silica particles were packed into a stainless steel column (5 cm, Ø 0.4 cm, 450 mg of catalyst material). The substrates and reagents were suspended and pumped through the packed-bed columns (containing the functionalized material) by an HPLC pump (Hitachi L-600, Merck). All reactions were carried out at room temperature. Aliquots were taken after different times (60/30/15/10/5 min) and different flow rates (0.05/0.1/0.2/0.5/0.75/1 mL min^–1^).

**PE**: The reaction solution consisted of 1.8 mL (15 mmol) of 1-phenylethanol, 3.12 mL (22.5 mmol, 1.5 equiv) of triethylamine, and 2.13 mL (22.5 mmol, 1.5 equiv) of acetic acid anhydride in 43 mL of toluene.

**TP**: For the flow-catalytic test, a solution containing 430 mg (1 mmol) of α-tocopherol dissolved in 49.5 mL of toluene was prepared. Then 277 μL (2 equiv) of trimethylamine and 189 μL (2 equiv) of acetic acid anhydride were added.

**Aliquote analysis**: The collected 1-phenylethanol aliquots were prepared for further analysis by adding 150 μL of the reaction solution to 750 μL of methyl *tert*-butyl ether (MTBE) and 15 μL of n-hexadecane (as internal standard) and analyzed by gas chromatography (Hewlett-Packard 5980 series, DB-WAX from Agilent).

Before further analysis, the gathered α-tocopherol aliquot was washed with distilled water (1:1), and 200 μL of the organic phase was added to 200 μL of acetonitrile. The yield was determined by HPLC (Dionex P680 pump combined with a Dionex UVD170 UV detector. For separation an Eurospher II CN column was used with a mobile phase consisting of 97% hexane and 3% iso-propanol at a flow rate of 1 mL min^–1^).

### Structural Characterization of Materials

Nitrogen physisorption experiments were performed with a Quadrasorb evo (Quantachrome Corporation) at 77 K. Argon physisorption data were recorded with an Autosorp iQ2 (Quantachrome Corporation) combined with a cryostat (Cryosync, Quantachrome Corporation) for temperature control at 87 K. Before physisorption measurement, the unfunctionalized material (393 K, 20 h) as well as the functionalized materials (373 K, 12 h) were degassed under reduced pressure. The collected data were evaluated using the “ASiQwin” software, version 4.0 (Quantachrome Corporation), using the available data reduction algorithms. For the analysis of the specific surface area the Brunauer–Emmett–Teller (BET) method was applied in the relative pressure range of *p/p*_0_ = 0.05–0.30. The calculated surface areas are consistent with nonlocal-density-functional theory (NLDFT) surface area values. The pore size distributions were derived from NLDFT kernels applicable for zeolites/silica for a cylindrical pore model with a moving point average (MPA) of 5. In the case of the functionalized materials, the physisorption data were normalized to silica mass, and not to the mass including the organic moeitites, for identifying the impact of the functionalization on the mesoporosity of the silica carrier material.

Mercury intrusion porosimetry was carried out in the pressure range 0–400 mPa (instrument: Pascal 140 and 440 porosimeter, Thermo Fisher Scientific).

For scanning electron microscopy, the materials were sputter-coated with platinum and measured with a Zeiss Merlin microscope (acceleration voltage of 3 kV and current of 90 pA).

A CHN-analyzer Flash EA-1112 instrument (Thermo Scientific) was used for elemental analysis.

Nuclear magnetic resonance (NMR) spectra (^1^H and ^13^C) were measured with a Bruker Advance II 400 MHz instrument at 298 K.

Diffuse reflectance infrared Fourier transform spectroscopy (DRIFT) spectra were recorded with a Bruker alpha instrument in the range of 400–4000 cm^–1^ and a resolution of 2 cm^–1^.

### Sample Preparation for Diffusion Measurements

Samples for diffusion measurements were prepared as saturated particles, i.e., with the pore volume of the unfunctionalized silica particles completely filled with liquid, while no excess liquid is left outside the particles. The liquids used were 1-phenylethanol (**PE**/0.3 mol L^–1^), phenylethyl acetate (**PEA**/0.3 mol L^–1^), α-tocopherol (**TP**/0.02 mol L^–1^), and tocopherol acetate (**TPA**/0.02 mol L^–1^) in toluene-d_8_. To achieve complete pore filling, the respective silica particles were weighed into NMR tubes (amount on the order of 100 mg), and a volume of liquid, corresponding to the pore volume of the particles as determined from Table 1, was added on top of the particle bed via a micropipette. After addition of the liquid, the sample was centrifuged at 4000 rpm for 10 min to force all liquid into the silica, and it was given 7 days at RT for all liquid to diffuse into the pores. It was checked by weighing before and after the 7 days that no liquid left the NMR tubes. A sample before centrifugation and after the 7 day waiting period is shown in the Supporting Information (Figure S11). The sample preparation was performed without prior drying of the silica particles. Drying of the silica prior to pore filling has an effect on the diffusion coefficients, as can be seen in the Supporting Information (Figure S12). However, drying was avoided in order to best represent the flow catalysis conditions.

**Table 1:** Properties of the Functionalized and Pure Materials^a^

Mesopore diameter	6 nm	10 nm	30 nm
*S*_BET unfunct._/m^2^ g^–1^	670	340	147
*S*_BET Azide._/m^2^ g^–1^	406	328	114
*S*_BET DMAP._/m^2^ g^–1^	350	290	100
*V*_pore unfunct._/cm^3^ g^–1^	0.75	1.18	1.07
*V*_pore Azide._/cm^3^ g^–1^	0.53	1.04	0.68
*V*_pore DMAP._/cm^3^ g^–1^	0.50	0.98	0.63
Azide loading/mmol g^–1^	0.47 ± 0.14	0.29 ± 0.09	0.32 ± 0.10
DMAP loading/mmol g^–1^	0.26 ± 0.07	0.26 ± 0.08	0.18 ± 0.05

a BET surface area and pore volume (derived from N_2_ physisorption, *T* = 77 K) of the three types of spherical silica particles (5 μm diameter for each type of particle) possessing an average mesopore diameter of 6, 10, and 30 nm, before and after functionalization with the azide group as well as DMAP organocatalyst, and corresponding loading.

### Diffusion Experiments

All PFG NMR measurements were performed at 25 °C on a 400 MHz spectrometer (Avance III HD, Bruker), equipped with a gradient probe head (Diff50, Bruker) with a selective ^1^H insert, and providing magnetic field gradients up to 28.5 T m^–1^. To calibrate the temperature, a PT 100 thermocouple, inserted into an oil-filled NMR tube, was used. Diffusion measurements were conducted by a stimulated echo pulse sequence (Figure S15), taking a series of spectra with a variation of the gradient pulse strength *g*. The maximum gradient pulse strength *g* (up to 25 T m^–1^) and the gradient pulse duration δ (up to 2 ms) were adjusted for each sample. The time between the first 90° pulses, τ_1_, in which the gradient pulse is applied, was at most 0.8 ms longer than δ. The observation time Δ was kept as short as possible (Δ = 6 ms, if not mentioned otherwise). If not mentioned otherwise, the resulting echo decay intensity *I* was evaluated by the Stejskal–Tanner equation^46^
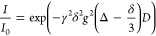
1in which γ denotes the gyromagnetic ratio of the ^1^H nucleus.

Various models, taking into account the complexity of the porous materials, which deliver echo decays beyond the single exponential of eq 1, are discussed in the Results and Discussion. To verify the model chosen, besides the silica particles with a diameter of 5 μm, an additional batch of LiChrospher Si100 with a particle diameter of 10 μm was employed (see Figure S5 for structural characterization).

## Results and Discussion

The different particles were thoroughly characterized by nitrogen and argon physisorption (*T* = 77 and 87 K, respectively). Out of the three chosen mesopore sizes, the particles with an average mesopore diameter of 30 nm possess the smallest BET surface area of 147 m^2^g^–1^, which only allows for a moderate loading with the organocatalyst and thus constitutes a limiting parameter for catalyst immobilization (Table 1).

**Figure 2:**
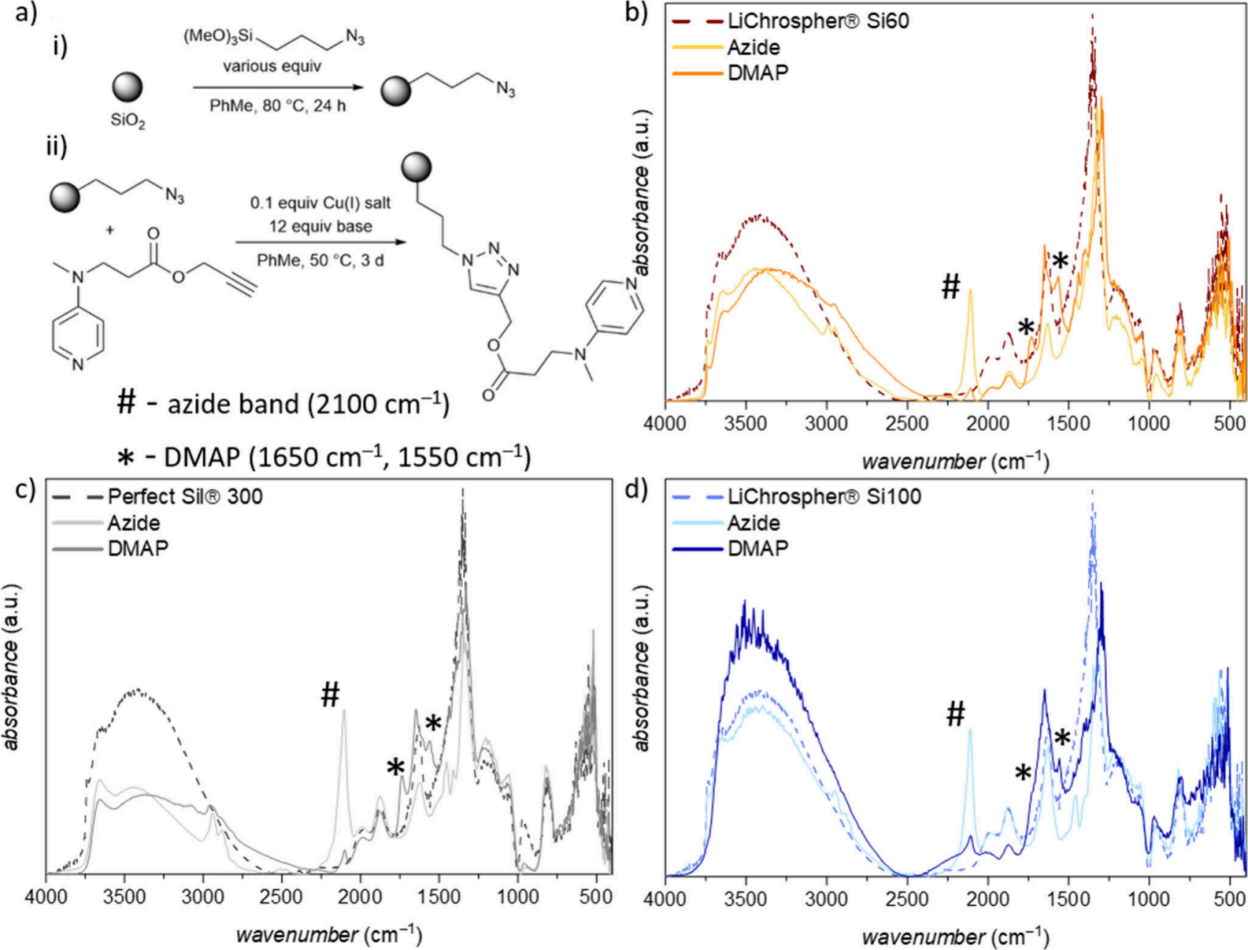
**a** Reaction scheme for the immobilization of the DMAP derivate onto porous silica. **b-d** show the DRIFT spectra of the unfunctionalized state and both functionalization steps for each material where the #- labeled bands correspond to the azide group, and the *-labeled signals to the DMAP catalyst.

Using an efficient and reliable immobilization strategy consisting of an alkyne–azide-based click-chemistry approach, we were able to immobilize 0.2 mmol g^–1^ of DMAP derivative in the 30 nm-mesopore-material (Figure 2).^21,47^ The loadings of the 10 nm (340 m^2^g^–1^ BET surface area) and 6 nm (670 m^2^g^–1^ BET surface area) materials were adapted to fit this loading value, thus resulting in three materials with comparable catalyst loadings, if normalized to mass. Note that preparing materials with similar surface coverage, i.e., defined as amount of catalyst normalized to the surface area, cannot be achieved, as already described in our recent study.^17^

The amounts of functionalized linker and catalyst were calculated based on the nitrogen content, which was quantified by elemental analysis. A detailed calculation of the catalyst loading as well as the corresponding elemental analysis data are shown in the Supporting Information file (equations (S1) to (S3) and Table S1). When the achieved loading is compared with the azide group and the DMAP catalyst, it is noticeable that the mesopore volume decreases more significantly for the first grafting step and that a higher quantity of the azide linker is immobilized compared to the subsequent DMAP coupling.

For studying the immobilization steps, DRIFT spectroscopy for each functionalization step was performed (Figure 2b-d). The introduced azide linker shows a characteristic IR-band around 2100 cm^–1^ whose intensity decreases upon the subsequent click reaction with the DMAP derivate. In the spectra of the fully functionalized materials, two bands at 1650 cm^–1^ and 1550 cm^–1^ appear, which correspond to C=C and C=N stretching vibration of the pyridine ring.^44^

### Physisorption Analysis

**Figure 3:**
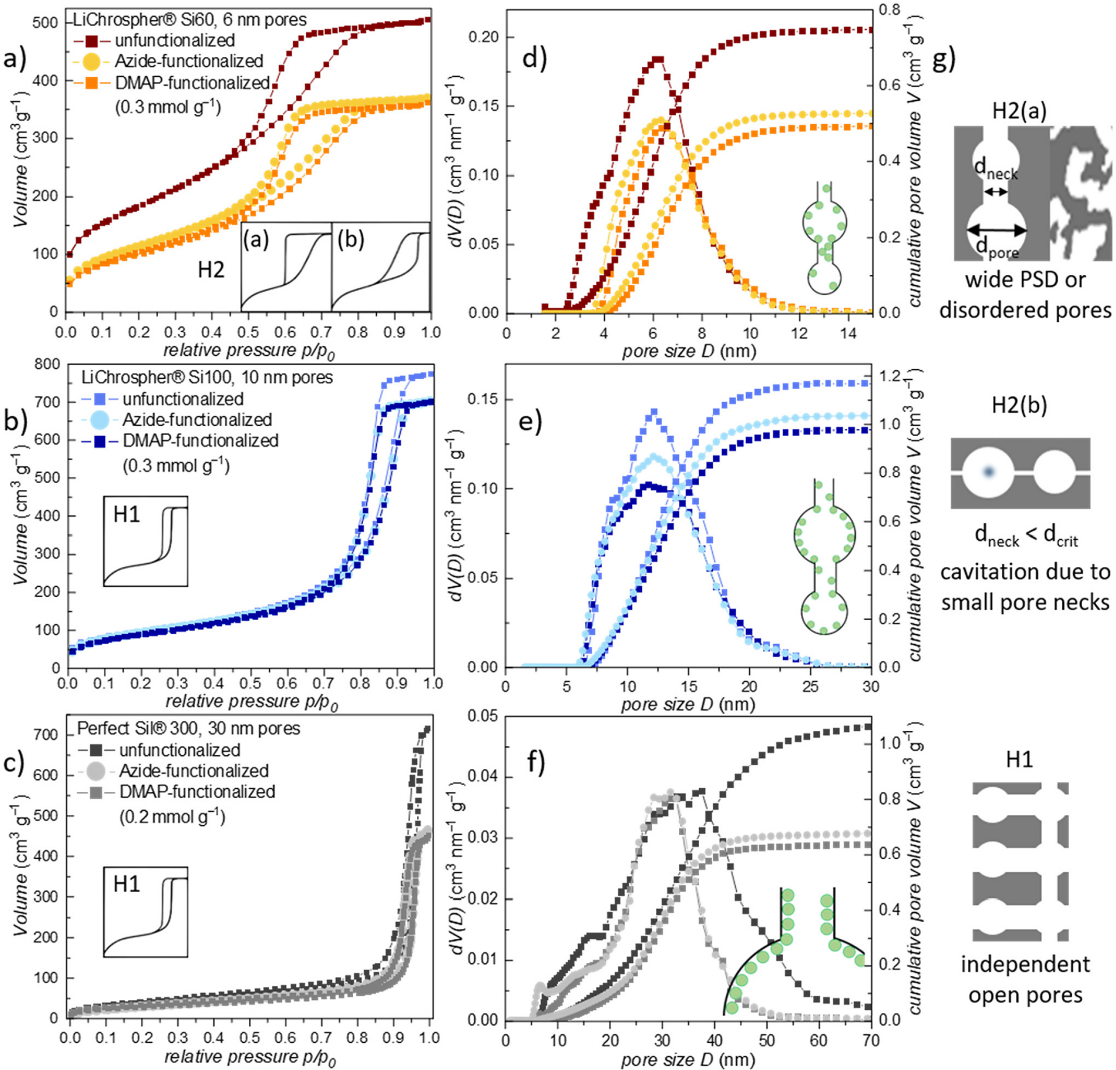
**a**-**c** Impact of DMAP immobilization on physisorption isotherms and **d**-**f** differential and cumulative PSD (N_2_, 77 K, NLDFT model for zeolites/silica, cylindrical pores, adsorption branch) for each of the three types of silica particles. The physisorption isotherms of the raw and azide-functionalized materials are depicted in Figure S6 in the Supporting Information. Panel **g** shows schematics of the IUPAC classification for different hysteresis types and the assigned pore networks (reproduced from Schlumberger et al., copyright 2021 Wiley VCH GmbH).^48^

Physisorption is a versatile method for the characterization of mesoporous materials, which gives not only typical porosity parameters such as internal surface area and pore size distributions (PSD), but as well qualitative insight into the mutual pore connectivity.^48,49^ Notably, for diffusion of molecules into mesopores, not only the average pore diameter but also the connection to adjacent mesopores is relevant. For mesopore networks, different desorption mechanisms are generally possible in the case of hindered pore connection, resulting for instance in “pore blocking” effects or “cavitation” phenomena. Pore blocking occurs when pore entrances or connecting pores are markedly smaller than the diameter of the larger mesopores and is often combined with H2(a) or H2(b) hysteresis (Figure 3g). In these “bottle-neck” pores, the desorption-branch-derived PSD is shifted to smaller values because the pressure of evaporation is dependent on the neck size rather the actual mesopore size. When the diameter of the connecting pore is below an adsorptive-dependent critical diameter, a gas bubble is formed in the liquid upon reducing the pressure. This process is called cavitation and associated with H2(a) or H1 hysteresis.^50^

As a consequence of the presence of “pore blocking” or “cavitation”, meaningful PSDs can only be obtained from the adsorption branch of isotherms, using suitable NLDFT analysis.^33,34^

Figure 3 shows the N_2_ physisorption isotherms and the corresponding PSDs, determined from the adsorption branch via NLDFT analysis, performed on data of the unfunctionalized material, and the azide- and DMAP-functionalized materials (see Figure S7 for additional fitting errors for the NLDFT-kernel). All materials show isotherms that match the IUPAC-classified type IV(a) for mesoporous materials but exhibit different hysteresis types. A close look at the raw physisorption data shows that the azide- and DMAP-functionalized isotherms are almost overlaying exactly, especially for the 10 nm material (Figure 3b, c). In the case of the 6 nm material (LiChrospher Si60), the shape (Figure 3a) can be assigned to type H2(a) or H2(b) and thus indicates hindered evaporation from the mesopore network resulting in pore-blocking effects or cavitation. The corresponding PSD (Figure 3d) exhibits pore diameters between 3 and 14 nm with a small but significant shift to larger values after functionalization, presumably originating from clogging/filling of the smallest mesopores and necks by the immobilized catalyst. Both 10 nm (LiChrospher Si100) and 30 nm (PerfectSil 300) materials demonstrate a type H1 hysteresis with a broad PSD (Figure 3b, c, e, f). In both cases, the PSDs remain almost unchanged upon functionalization. Since the PerfectSil 300 isotherms indicate mesopores larger than 50 nm, the obtained PSD was verified by Mercury Intrusion Porosimetry (Figure S4). Upon functionalization, a substantial decline in the mesopore volume and absorbed volume is visible in Figure 3d-f, corresponding to the catalyst taking up space inside the mesopores. While the first grafting step shows a higher impact on the mesoporosity regarding PSD and surface area, the shape and type of the hysteresis remain unchanged and hence indicate an even distribution of the azide linker on the surface of the materials. The immobilization of the final DMAP catalyst only leads to a small decrease in the surface area and mesopore dimension, indicating that a certain fraction of the azide groups is still present after the click reaction, possibly due to steric hindrance and diffusion limitation. Thus, for both materials, the evaluation of the physisorption data suggests that the catalyst covers the pores’ surface without occurrence of mesopore clogging. Yet, there is no change in the shape of the isotherms and thus the hysteresis types, suggesting the absence of functionalization-induced evaporation hindrance such as pore-blocking or cavitation, while such a conclusion is ambiguous if only based on one adsorptive (here N_2_). Building on a recently summarized procedure, we thus additionally calculated the PSDs from an adsorption and desorption branch of Ar (87 K) physisorption isotherms (Figure S9), to further elucidate the connectivity between mesopores, especially to find signs of pore blocking, i.e., the presence of a pore entrance or connecting pores being markedly smaller than the diameter of the larger mesopores.^48,49^ The PSDs determined from Ar (87 K) and N_2_ (77 K) physisorption show only a negligible content of micropores and small mesopores. Also, the close similarity of the PSDs calculated from the respective desorption branches of the two fluids (Figure S9) excludes the cavitation phenomenon according to a recently described procedure.^48,51^

**Figure 4:**
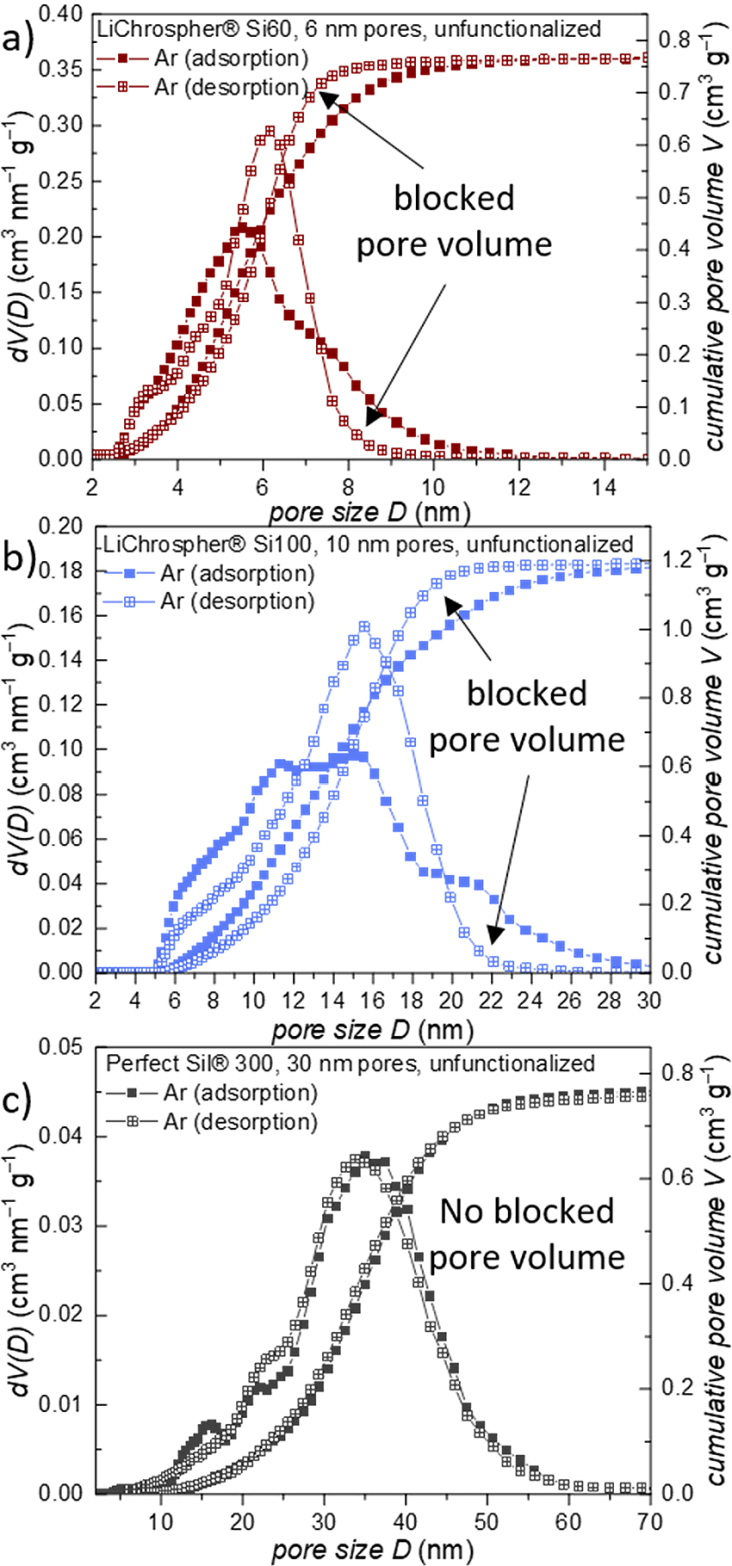
Comparison of adsorption and desorption branch derived differential and cumulative PSD (Ar 87 K, NLDFT for zeolites/silica, cylindrical pores, MPA of 5), which proves pore-blocking effects for **a** 6 nm, **b** 10 nm, and **c** 30 nm unfunctionalized materials. The corresponding Ar isotherms are shown in Figure S8.

Interestingly, for the unfunctionalized materials possessing 6 and 10 nm pores, the PSDs determined from the Ar adsorption and desorption branches (Figure 4) are substantially different, especially the desorption-based PSDs are narrower than the adsorption-based PSDs. Following refs (48, 49), this difference indicates pore blocking for the respective larger pores, i.e., connection of these pores through smaller neck pores.^48,49^ For the 6 nm porous material, this finding implies that mesopores larger than ca. 8 nm are connected through smaller pores, probably being on the order of 3 nm in diameter. The same effect can be found for pores larger than about 21 nm in the 10 nm mesopore material. Since the NLDFT fit is relatively poor in the lower relative pressure range, the evaluation of the small mesopores under 6 nm should be assessed carefully (see Figure S7).

**Figure 5:**
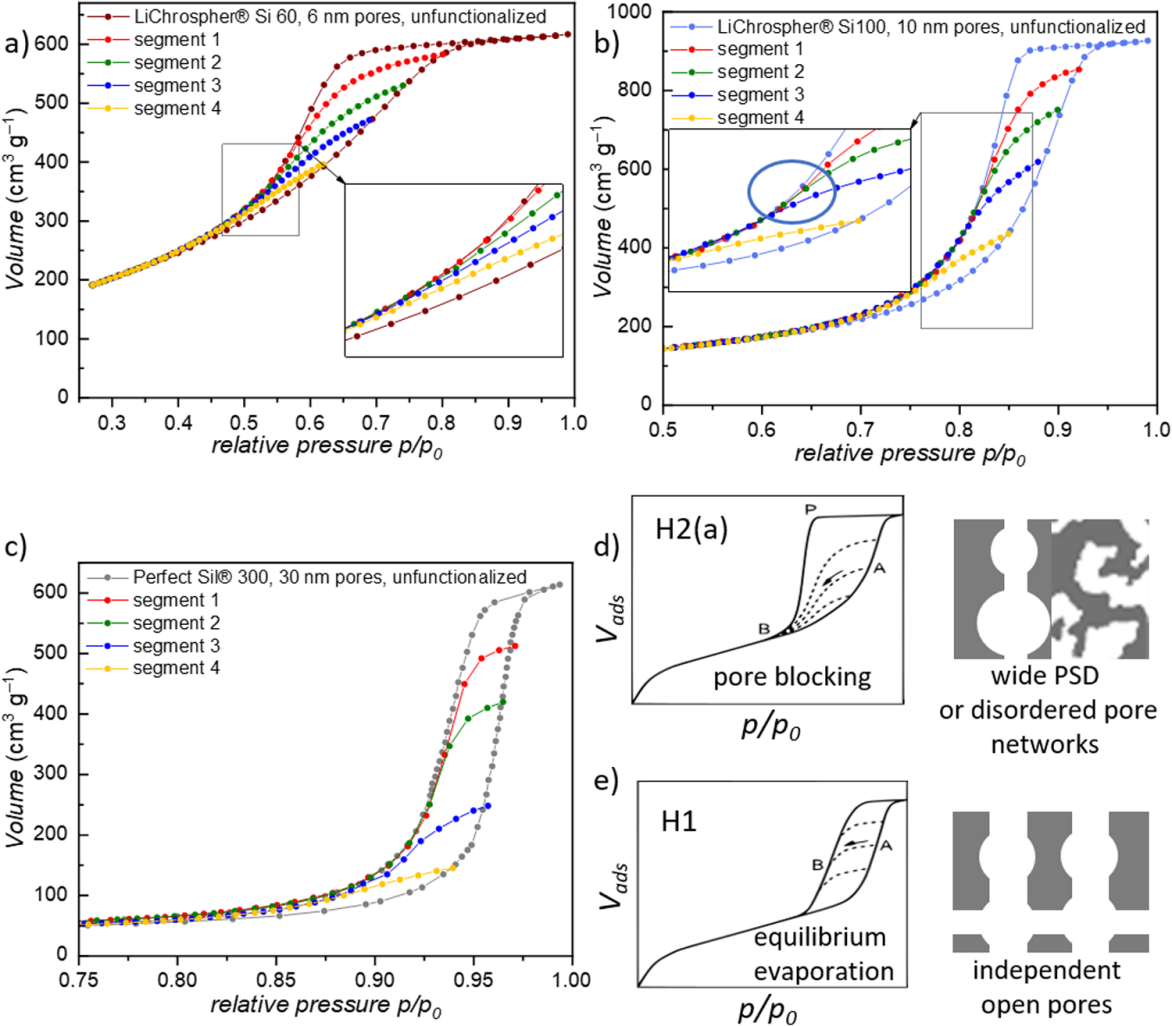
**a-c** Hysteresis desorption scanning curves with four different desorption branches (the adsorption branches are not depicted for visual clarity, for complete data check Figure S10 in the SI) for each material (using Ar, 87 K). **d-e** For better comparison, a schematic illustration of the occurring hysteresis and the corresponding desorption isotherms is shown (panels **d-e** were reproduced from Kube and Turke et al.,^50^ Copyright 2020 American Chemical Society).

In order to get further insight into the connectivity of the mesoporous network, hysteresis scanning curves (HSC) with argon as an adsorbate (87 K) were recorded (Figure 5). By measuring multiple desorption isotherms, each starting at decreasing relative pressure points, different filling states of the porous systems are achieved, which thus correspond to different populations of pore sizes. The shape of these scanning isotherms is specific to the different desorption mechanisms as well as the underlying pore connectivity. For the 6 and 10 nm porous materials, it is seen (Figure 5a, b) that the desorption isotherms meet the enveloping isotherm in one single point, at a lower relative pressure than expected for independent open mesopores. This observation implies that the depletion of larger mesopores depends on the filling state of smaller connecting pores. The overall shape of the multiple hysteresis curves indicates that the larger mesopores are connected by smaller mesopores, inducing pore blocking effects, thus confirming the aforementioned PSD-based analysis. Also, the mesopore space of the 10 nm porous material contains smaller, independent mesopores because for the last measured desorption cycle the curves coincide before the closing point of the envelope. Contrary to the 6 and 10 nm pore materials, the 30 nm porous material̀s desorption isotherm and the HSC data (Figure 5c) are equivalent to a H1 hysteresis type and therefore are in agreement with an open and independent pore network.

### Catalysis

The catalytic efficiency of the immobilized DMAP catalyst was evaluated by performing experiments under batch and flow conditions. For meaningful comparison, the measured conversions were converted to their respective turnover frequencies (TOF) according to eq 2 to account for small differences in catalyst loading.^21^
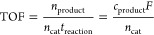
2where TOF is the turnover frequency, *n*_product_ the amount of product, *n*_cat_ the amount of catalyst, *t*_reaction_ the reaction time, *c*_product_ the concentration of product, and *F* the flow rate.

**Figure 6:**
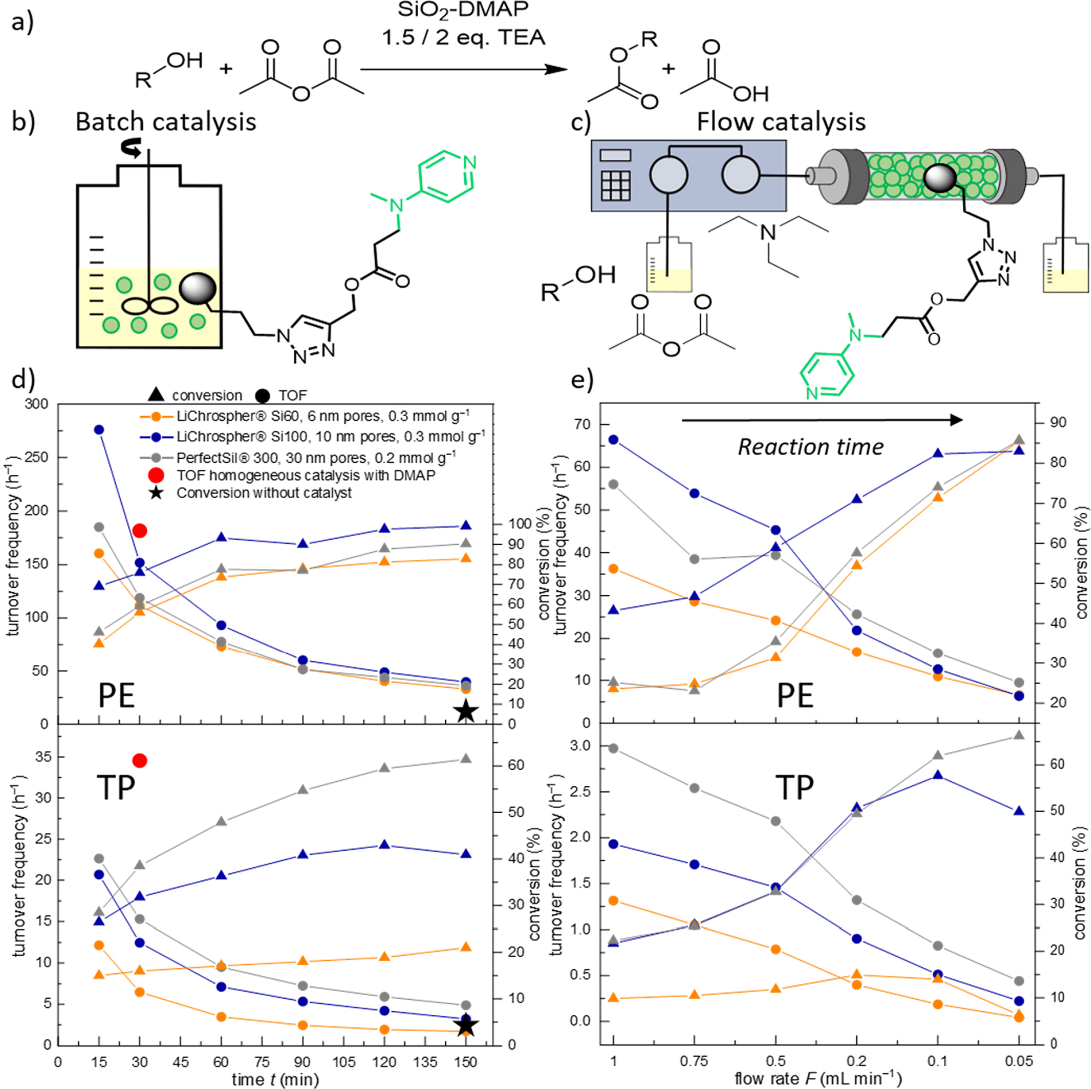
**a** Scheme of the catalyzed reaction, **b**, **c** Setup for batch and flow catalysis, **d, e** conversion values and the corresponding turnover frequency **d** for batch and **e** for flow catalysis of 1-phenylethanol (**PE**) and α-tocopherol **(TP**). Please note that for flow catalysis, the exact reaction times are not shown here, because they are not just the mere time for flow-through but need to be calculated, based on the overall porosity of the carrier material. Yet, the qualitative overall trend is obvious, i.e., with decreasing flow rate the reaction times increase (for a detailed description for the reaction time calculation, see Equation (S13)).

For catalytic tests in flow, the functionalized silica particles were packed into a stainless steel column (5 cm, Ø 0.4 cm, 450 mg catalyst material). The reaction solution containing either **PE** or **TP** was pumped through the column at various flow rates, and aliquots were collected and analyzed. Overall, the conversion of **PE** and **TP** into the respective acetylated product was quite significant for all materials, while the uncatalyzed reactions show conversions under 7% after 150 min (Figure 6d, star symbol).

### Batch Catalysis

In the batch experiments, the material was directly mixed with the reagent solution and the reaction was carried out under rapid stirring (Figure 6b). The shape of the silica particle was stable under the selected conditions. As illustrated in Figure 6d, all materials show an increased conversion with rising time, while the TOF values decrease due to the declining substrate concentration. In the acetylation reaction for **PE**, the 10 nm pores show complete conversion after 150 min, whereas the 6 and 30 nm pore particles achieved a conversion of over 80 and 90%, respectively. The particles possessing 10 nm pores exhibit the highest TOF values for the conversion of **PE.** However, quite a different trend is observable for the much larger **TP** molecule: here the material with 30 nm pores outperforms the other two particle types and reaches conversions of about 60% after 150 min, followed by the 10 nm pores, which reaches a conversion of about 40%, and a low conversion of around 20% for the 6 nm pore particle. However, the TOF values reveal that the performance of the 10 nm pores is close to that of the 30 nm pores, especially in the beginning of the reaction, which might be due to a trapping of **TP** or the acetylated product inside the pores. Although the 10 nm porous material seems superior in regard to catalyst loading, surface area, and mesoporous volume, we conclude that in terms of organocatalysis this pore size is only optimal for smaller molecules like **PE**, but insufficient for larger molecules like **TP**. In turn, the 30 nm pores seem to be advantageous for larger molecules such as **TP** in regard to diffusion/transport, and the unhindered connectivity (see physisorption) might compensate for the lower catalyst loading and smaller surface area, if compared to the 10 nm mesopore material.

In the case of the 6 nm pore particles, the comparably low catalytic performance can be attributed to hindered diffusion, this interpretation being backed by the pore-blocking effect observed in physisorption analysis, caused by smaller and poorly interconnected mesopores. Since the material is not actively flowed through under batch conditions, passive diffusion into the particles comes into full effect here. Thus, we conclude that the mesopores in the 6 and 10 nm particles are apparently too small and insufficiently connected for an efficient mass transport inside the particles. Also, it is likely that catalysis taking place on the outside of the particles is more relevant compared to the 30 nm pore particles, because of reactant or product molecules being trapped in the small mesopores (necks), an effect similar to membrane fouling.^52^ These effects lead to low conversions for these particles, while 30 nm pores allow the whole inner surface to participate in the reaction.

### Flow Catalysis

The evaluation of the flow catalysis data (Figure 6e) shows that the conversion rises with lower flow rates, which is in agreement with general principles of catalysis in that the contact time of the reactants with the reactor (and hence the reaction time) increases (the respective reaction times were calculated with Equation (S13)). For TOF values, concomitantly this trend is reversed: the lower relative conversion of high flow rates is more than compensated for by the higher mass flow of reactant molecules and thus the generation of more product in absolute terms. This effect vanishes with decreasing flow rate and eventually diminishes at very low flow rates like 0.05 mL min^–1^, because the catalysis conditions align with the diffusion observed in batch conditions since there is no pump-enhanced flow through the material. In contrast to batch conditions, which is governed by the self-diffusion to and within the porous particles, the transport inside and through a flow reactor is more complex, because the reaction mixture is actively pumped through the column, here by an external HPLC pump. In liqud chromatography, the basic transport relevant contributions can be described by the van Deemter equation, where the transport is divided into (i) the dispersion of the molecules due to different pathways in the interparticle void (Eddy diffusion), (ii) diffusion leading to a longitudinal distribution and (iii) the mass transfer kinetics between the mobile and the stationary phase, which is determined by the mesopores in the silica phase.^53,54^ The underlying transport mechanisms in flow and batch conditions thus differ significantly and can therefore result in opposing trends in catalysis, especially for the larger molecule **TP**.

For the small molecule **PE** a similar trend as observed for batch catalysis is seen: the 10 nm pore material shows by far the highest values with a TOF of 65 h^–1^, while the 6 and 30 nm pore particles achieve similar conversions and TOF values around 45 h^–1^ and 55 h^–1^ respectively, while the numbers equalize with slower flow rates. For comparison, a commercially available silica-bound DMAP catalyst reached a TOF of approximately 28 h^–1^ for a Morita–Baylis–Hillman reaction in a study of Verdier et al., while our reactions reached values between approximately 1 and 100 h^–1^.^55^ Although the reaction is very different from that of acylation, this shows that our material is capable of producing commercially competitive TOF values.

Interestingly, in the case of **TP**, the opposite trend is noticeable: the largest pore size of 30 nm gives results similar to the 10 nm pores for high flow rates, though the conversion observed for the 10 nm pores shows a steep decline with low flow rates indicating slow diffusion of the **TP** inside the flow reactors. Under consideration of the van Deemter equation, this behavior indicates that for both the 10 and 30 nm materials, the mass transfer kinetics of the reactants into the porous particles is similar. For all flow rates, the material with 6 nm pores shows the smallest TOF values. Thus, while already for a small molecule as **PE** diffusion into and within the 6 nm pores is hindered, consequently the larger molecule **TP** exhibits the smallest TOF values also for the 6 nm mesopores. Summing up, for both reactants, the largest mesopore size (30 nm) gets more favorable the slower the flow rate is, presumably, because the longer contact time rates leave more time for self-diffusion through the reactor and into the mesopores of the carrier material. Thus, the self-diffusion being the relevant transport mode at small flow rates resembles the conditions of the batch experiments, and therefore, the 30 nm pore particles exhibit enhanced catalytic performance. The geometric dimension of **TP** can be estimated to approximately 1 nm, although the hydrodynamic dimension might differ in solution.^51,56^ While it is evident that a 6 nm pore might inhibit the intrusion and diffusion of a 1 nm-sized reactant, the experiments reveal a distinct difference between the 10 nm vs 30 nm mesopore material, which is surprising if only the mere pore size is considered. Overall, markedly higher productivity is achieved with **PE** – the TOF values differ by approximately a factor of 10 compared to **TP**. The comparison between the TOF values obtained from homogeneous and heterogeneous catalysis shows that the esterification of both alcohols **PE** and **TP** proceeds on the same order of magnitude, implying that the mass transfer limitation is similar and small. Table 2 summarizes the calculated TOF values of free DMAP and immobilized DMAP after 30 min reaction time. Since the reaction time for the continuous setup corresponds to the residence time of the reaction solution inside the reactor (calculated according to equation (S13)), the TOF values are not as comparable as data obtained under batch conditions.

**Table 2:** Calculated TOF Values for Homogeneous and Heterogeneous Catalysis under Flow and Batch Conditions for PE and TP

		Material	DMAP (homogeneous)	6 nm	10 nm	30 nm
PE	Batch	TOF/h^–1^	181.6	111.6	151.6	118.7
		*t*_reaction_/min	30	30	30	30
	Flow	TOF/h^–1^	-	36.3	66.5	56.0
		*t*_reaction_/min	-	0.8	1.0	0.9
TP	Batch	TOF/h^–1^	34.6	12.2	20.7	15.3
		*t*_reaction_/min	30	30	30	30
	Flow	TOF/h^–1^	-	1.3	1.9	3.0

Since in homogeneous catalysis both reactants react rapidly (see Figure 6d, red dots), for all three mesopore sizes diffusion of **TP** into or inside the mesopores within the particles, where the majority of catalyst is immobilized, is hindered. For a detailed analysis of the diffusion behavior of the substrates in the different materials, we performed PFG NMR experiments for clarification; see further below.

**Figure 7:**
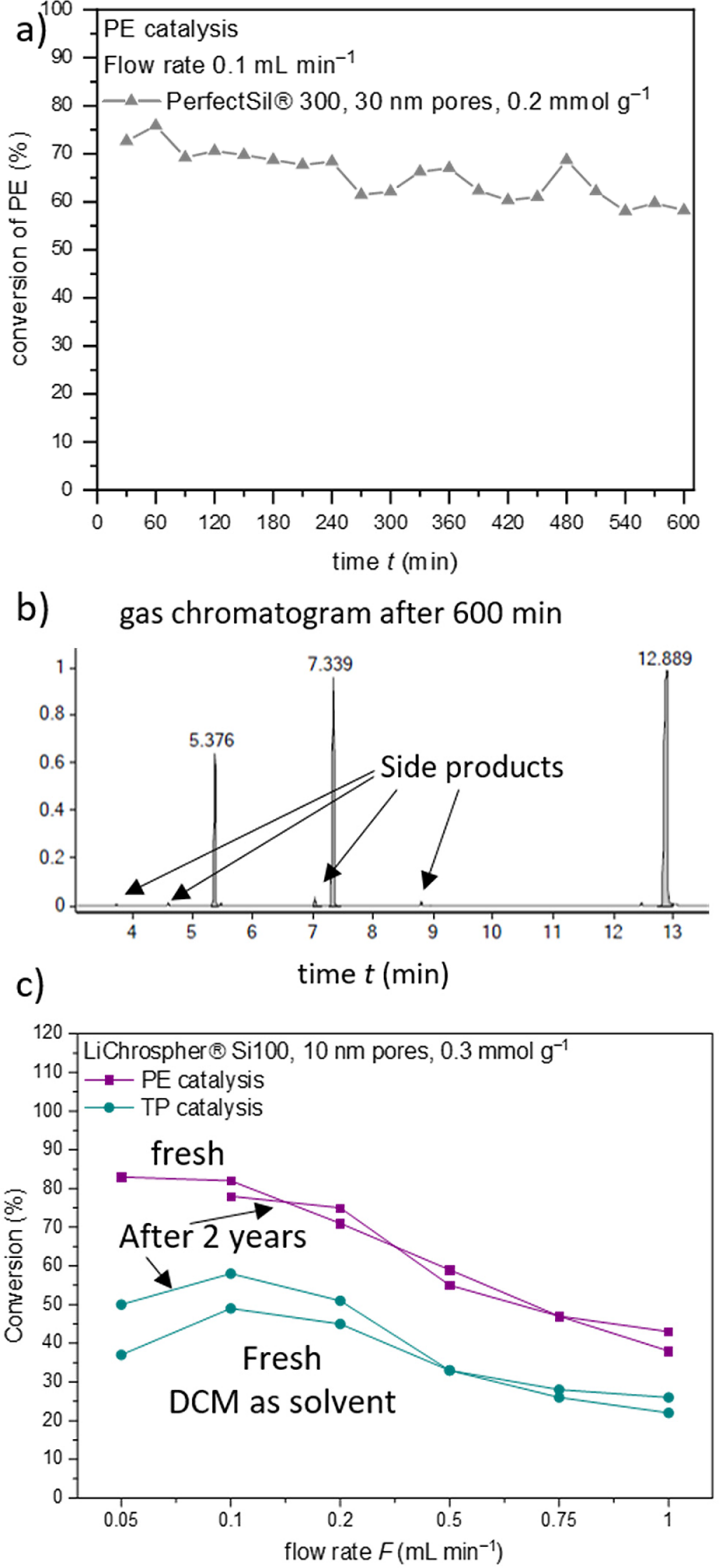
**a** The materials show a constant conversion of PE even after 10 h of constant use, but **b** shows that in the corresponding gas chromatogram, additional peaks are forming due to decomposition of acetic acid anhydride. **c** Even after 2 years, the materials are still catalytically active in the esterification of PE and TP, and the deviation of TP conversion is due to different solvents.

Concerning recyclability and long-term stability, the catalytic stability under flow conditions showed a relatively stable conversion over 10 h (Figure 7a). After 5 h, additional peaks in the GC chromatogram in Figure 7b appear, but they diminished after the reaction solution was changed. This indicates that these side products are forming in the reaction solution itself and not appearing due to change of the catalytic materials. As these side products are most likely due to decomposition of the acetic acid anhydride under basic conditions, the anhydride should be added to the reaction mixture separately. As depicted in Figure 7c, both alcohols **PE** and **TP** show a stable and reproducible conversion under the applied flow catalysis conditions. The experiments of each alcohol were performed at intervals of 2 years, demonstrating that the catalyst is quite stable on the silica surface.

### PFG NMR Diffusion Experiments

**Figure 8:**
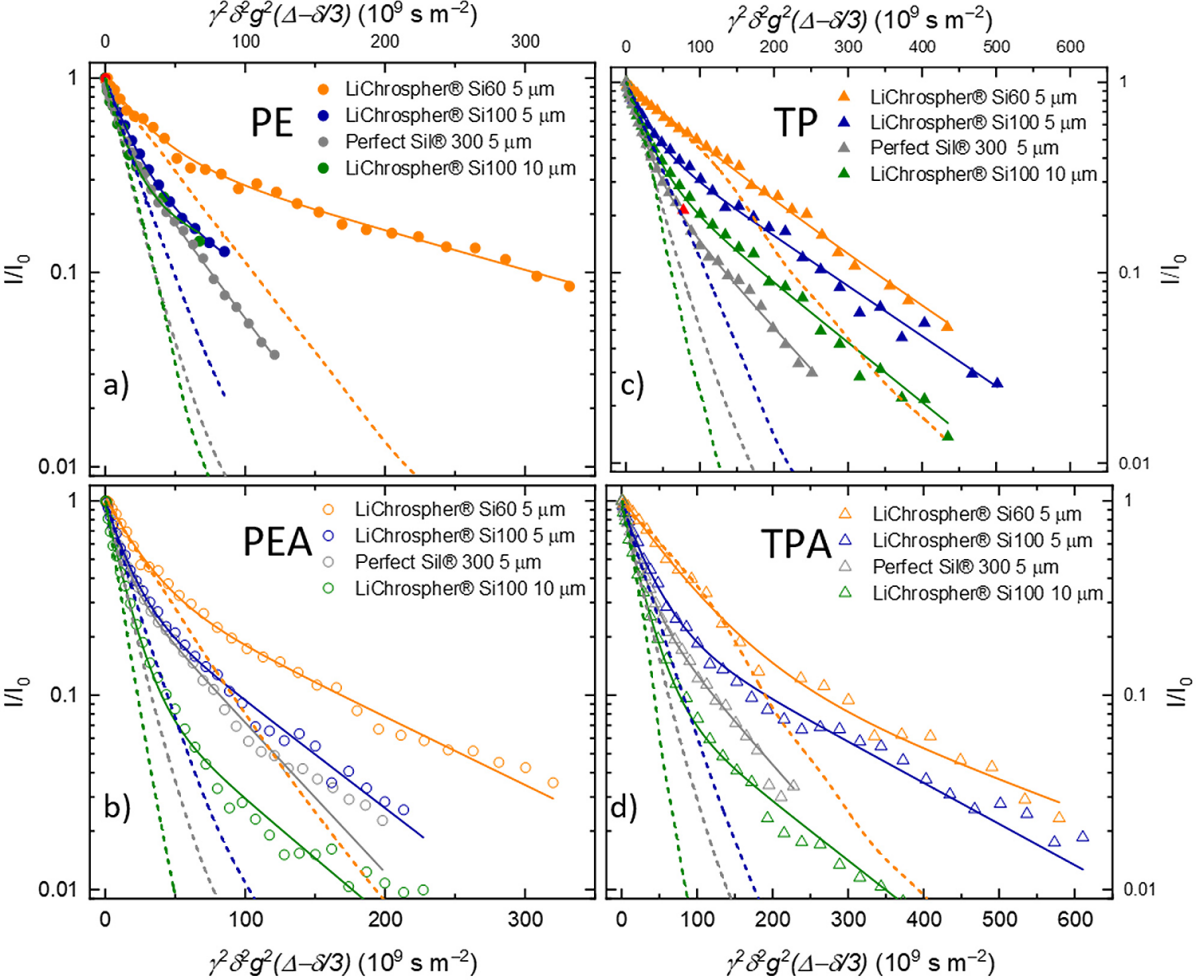
PFG NMR analysis: diffusion attenuation curves of **PE a**, **PEA b**, **TP c** and **TPA d** in LiChrospher Si60 5 μm (orange), LiChrospher Si100 5 μm (blue), LiChrospher Si100 10 μm (green) and Perfect Sil 300 5 μm with a biexponential fit (solid lines) and a fit of the initial signal attenuation with the model of restricted diffusion in a sphere according to Balinov et al.^57^ (dashed lines).

In Figure 8, the diffusion attenuation of phenylethanol **PE a**, phenylethyl acetate **PEA b**, tocopherol **TP c** and tocopherol acetate **TPA d** in the porous materials is shown, plotted on a logarithmic scale.

It can be clearly seen that the curves deviate from an exponential decay. This discrepancy is in contrast to the decay curves of the same liquids in a nonconfined state, see Figure S13, where an exponential decay allows the extraction of a well-defined bulk diffusion coefficient (Table S2) according to eq 1. A deviation from an exponential decay in porous materials can be caused by several effects,^27,30,57−60^ which we discuss in the following in order to apply a suitable model, which allows the extraction of the internal diffusion coefficients *D*_eff_ within the particles.

**Figure 9:**
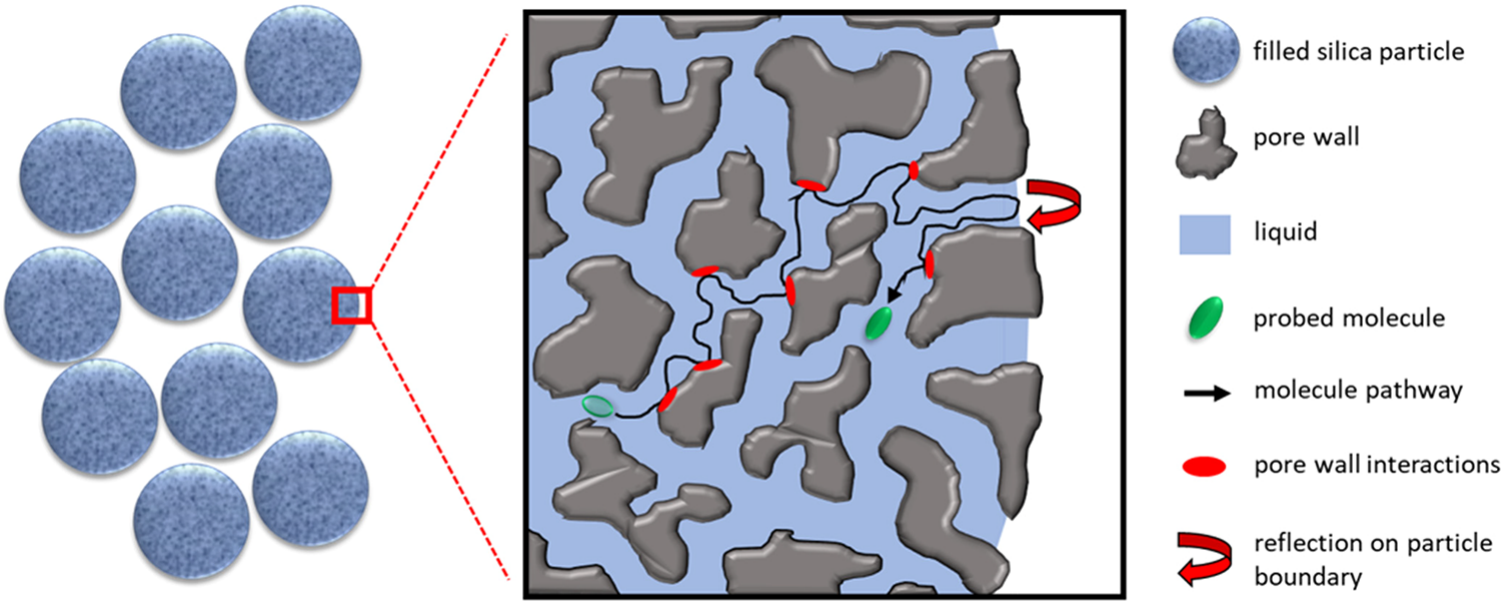
Sketch of the diffusion pathway of a molecule in a porous silica particle.

First of all, it is important to note the time scale of the experiments and the associated mean square displacement, which is probed. The mean square displacement of a molecule confined in the porous materials can be estimated for 3-dimensional diffusion according to ⟨*R*⟩^2^ = 6*DΔ*. With the observation time of Δ of 6 ms and values for the confined molecules of *D* of around 1 × 10^–10^ m^2^ s^–1^, the estimated root-mean-square displacement would be 2 μm. This is far larger than the mesopore diameter of the silica but is on the order of the particle radius. Thus, on the one hand, the diffusion coefficient is determined as a population average over molecules with different local positions, such as adjacent to a pore wall versus in a pore center and similarly over larger or smaller pores. On the other hand, there is no macroscopic averaging beyond the scale of 2 μm and in contrast to molecules around the center of the particle, those further outside may exhibit different displacements, as they are affected by the particle boundary. Note that for avoiding exchange with molecules in the interparticle voids, which would prevent the extraction of intraparticle diffusion coefficients, we have purposefully used saturated particles with no excess liquid in the interparticle voids; thus, the diffusion pathways are restricted to the particle interior, as displayed in Figure 9. In general, a deviation from exponential echo decay curves in a diffusion experiment may arise from different effects, which we carefully discuss in the following (see also the extended discussion in SI), in order to identify a suitable model for fitting the data to extract the intraparticle diffusion coefficient.

Deviations of the echo decay from an exponential behavior may arise from (i) a true distribution of diffusion coefficients, (ii) artifacts due to internal magnetic field gradients, or (iii) geometric constraints of the confinement inside a particle.

The first case (i) could be for example caused by “bottle-necks”,^27^ where some molecules may become trapped in narrow pores, possibly aided by strong interactions with the pore walls,^60^ and therefore display slower diffusion coefficients. In case that the exchange between the species (time scale τ_ex_) is sufficiently slow, i.e., τ_ex_ ≫ Δ, no averaging during the observation time Δ occurs and different species are observed as a superposition in the echo decay. The case of a broad distribution of the properties of trapped states or, similarly, a heterogeneity of pore geometries might then lead to a distribution of diffusion coefficients, manifested as a nonexponential echo decay. In general, heterogeneity can be analyzed by fitting a distribution of diffusion coefficients to the echo decay.^61^ In catalysis applications, however, it is the fast diffusing components, which are most relevant, even if single molecules might exhibit a long lifetime in small pores acting as “bottlenecks”, see also the detailed discussion in SI in context of Figure S14. There, we argue that a long-range heterogeneity of pore sizes over an order of 400 μm, which would be required to explain the nonexponentiality in Figure 8, is extremely unlikely.

A further influence on the echo decays (case (ii)) may arise from internal magnetic field gradients, which are a particular challenge in studying porous materials. Due to different magnetic susceptibilities of the silica material, the pore-filling fluid, and the interstitial voids, internal magnetic field gradients persist in the sample. Their interference with the externally applied gradients affects the echo intensity, see a detailed theoretical treatment by Zheng and Price,^58^ and the theoretical section in the SI. There, we analyze the magnitude of the internal gradients based on line width determination (Table S3) and Equations (S6) and (S7). We furthermore discuss their influence based on Δ-dependent diffusion data (Figure S16) and conclude that the internal static gradients are most likely the origin of the deviation of the echo decays from the initial slope at higher *g* values. We further conclude that, similar to a procedure employed in literature, a fit of the initial slopes yields the true diffusion coefficient.^30^

Concerning the third effect (iii), deviations from exponentiality in μm-sized particles may occur even in case of homogeneous diffusion with one distinct diffusion coefficient *D* and no background gradients, since the diffusion takes place in a closed sphere, with root-mean-square displacements (*rmsd*) leading out of the sphere being forbidden, see Figure 9. The fact that the liquid is restricted to the space inside the particles effectively limits the *rmsd* for molecules with a distance less than *rmsd* from the particle surface as molecules experience a reflecting wall. In the present case, the estimated *rmsd* is in the range of 2 μm, while the silica particles have radii of 2.5 or 5.0 μm, such that the influence is large. The true intraparticle diffusion coefficient in this case can be obtained using the model of restricted diffusion in a sphere according to Balinov et al.^57^ We explain this model in detail in the SI, section (iii), see Figure S17 and Equation (S8) to (S10). Finally, we fit the low *g* region of the echo decay curves by this model, thereby neglecting the high *g* region, which is affected by internal gradients. The resulting fit curves are displayed in Figure 8 as dashed lines, and the diffusion coefficients are given in Table S4.

In summary, although the diffusion attenuation curves may be influenced by all three effects, even in combination, we could employ measures to take the different influences into account. As the size of the silica particles is known, the effect of diffusion in a closed sphere (iii) can be evaluated with a suitable model,^57^ as detailed in the SI. Concerning “bottle-necks”, (i) the fast component, given by the initial slope of the echo decays, is most relevant, as it reflects the fastest diffusing fraction. Furthermore, the magnetic field gradients (ii) influence the echo decay predominantly at high *g* values, as we discuss in the SI, section (ii). Consequently, in our strategy to approach the real diffusion coefficient as precisely as possible, we evaluate the internal diffusion coefficient from two different fitting procedures, both cases focusing on the initial slope to avoid the potential effect of trapped species on the one hand and internal gradients on the other hand. The model of diffusion in a closed sphere relies on just one parameter, the diffusion coefficient, which is adjusted to make the model curves fit the initial slope. The second approach is a simple biexponential fit, from which the fast-diffusing component is further evaluated, see Table S5. Both fits are displayed in Figure 8. The model of diffusion in a sphere could not fully describe the whole decay due to the additional influence of internal magnetic field gradients at higher gradient strength but takes the correct geometrical boundary conditions into account.

**Figure 10:**
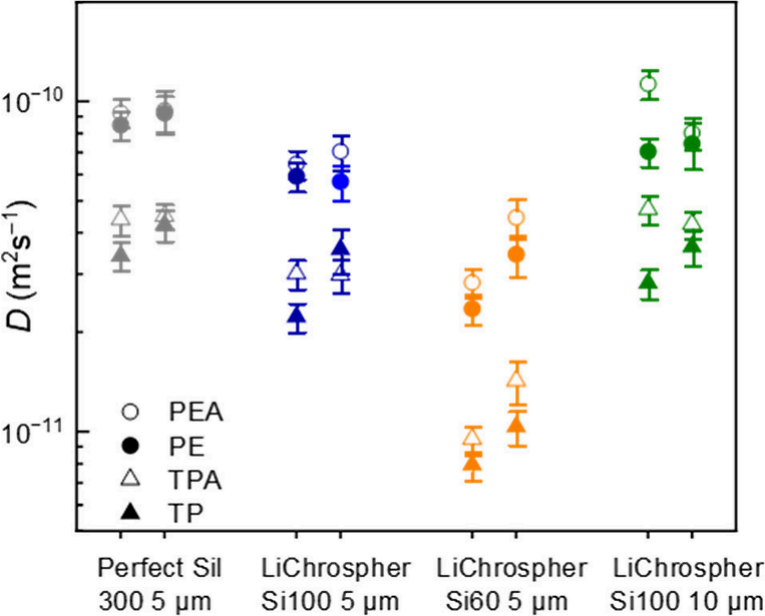
Self-diffusion coefficients of **PEA**, **PE**, **TPA** and **TP** in Perfect Sil 300, LiChrospher Si100 and LiChrospher Si60, obtained for a fit of the initial slope according to the model of diffusion in a closed sphere by Balinov et al.^57^ (left data points) compared to the faster diffusion coefficient of a biexponential fit (right data points).

In Figure 10, the diffusion coefficients of phenylethyl acetate **PEA**, phenylethanol **PE**, tocopherol acetate **TPA** and tocopherol **TP** in Perfect Sil 300, LiChrospher Si100 and LiChrospher Si60 are shown (see numerical values in Tables S4 and S5). The two models (respective of left and right data point) yield very similar results for the diffusion coefficients. The comparison of LiChrospher Si100 5 μm with the 10 μm sample serves as a validation of the chosen models. In these two samples with the same pore structure but different particle sizes, the effect of reflecting walls yields very different echo decay curves, but nevertheless, the resulting intraparticle diffusion coefficients are rather similar (compare green and blue data points). The detailed characterization by physisorption of both materials is depicted in Figure S5. We therefore concluded that our diffusion analysis procedure was valid and successful. In the following, we proceeded with the diffusion coefficients obtained from the reflecting sphere model because it uses only one fit parameter compared to the 4 fit parameters of the biexponential fit and is therefore more robust.

**Figure 11:**
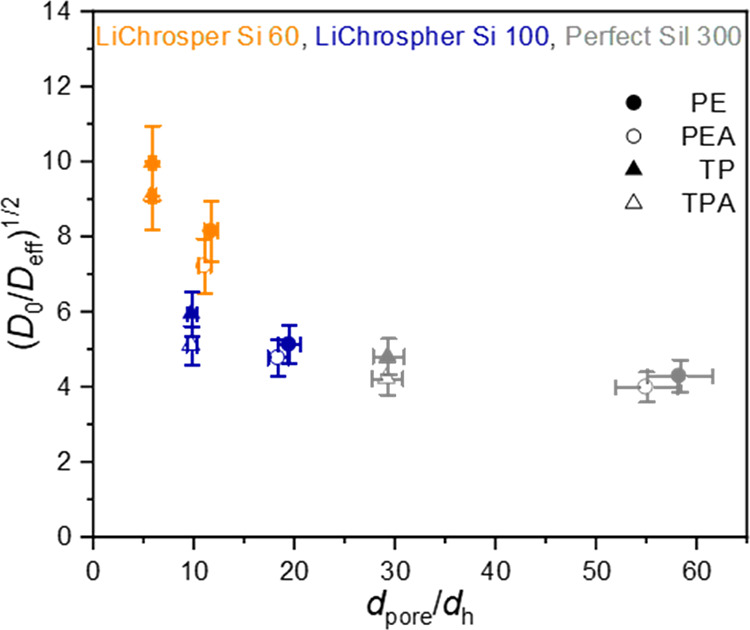
Plot of (*D*_0_/*D*_eff_)^1/2^ vs the ratio of silica mesopore diameter to hydrodynamic diameter of the respective molecule.

As can be seen in Figure 10, in all porous materials, the acetylated molecules have a higher or similar diffusion coefficient as compared to the respective alcohols, most likely due to reduced interactions with the silanol groups on the silica surface. Comparing Perfect Sil 300 (average pore size 30 nm) with LiChrospher Si 100 (average pore size 10 nm), the diffusion coefficients are only slightly reduced, compared to a more severe reduction for LiChrospher Si 60 (average pore size 6 nm). To highlight the different influences of geometrical and interactive restrictions on the transport in the porous materials, Figure 11 shows (*D*_0_/*D*_eff_)^1/2^, corresponding to the tortuosity, versus the ratio of the silica pore diameter to the hydrodynamic diameter of the respective molecule (Table S2).

There is no strict correlation between the pore-to-molecule size ratio and the values of (*D*_0_/*D*_eff_)^1/2^, which would be expected for a merely sterically hindered diffusion in nanopores according to the concept of tortuosity. The LiChrospher Si 60 samples display much higher values of (*D*_0_/*D*_eff_)^1/2^ than the other silica materials, even though the pore-to-molecule size is not always smaller. Therefore, a geometric size constraint is not the only quantity defining the transport. Studies of the pore size dependence of diffusion of liquids show that for small pores the interaction of the liquid molecules with the pore wall is the factor that dominates over geometric restrictions and defines the transport inside the porous material.^30,62^ This same behavior can be seen here. Perfect Sil 300 and even LiChrospher Si 100 seem to have sufficiently large pores to enable a transport that is not strongly restricted by liquid-surface interactions. This is in accordance with the pore size distribution, which showed no pores smaller than 6 nm for both silicas. On the other hand, the pores of LiChrospher Si 60 seem to be so small, with a major fraction of pores smaller than 6 nm (from pore size distribution evaluation), that here the liquid-surface interactions clearly dominate. Additionally, the effect of the interaction of the probe molecule with the pore walls in our study is magnified by the fact that all our probed species are dissolved at relatively low concentrations in a solvent (toluene-d_8_), which has a relatively low affinity to the silica pore wall. This may be the reason why even for larger pores relatively high values of (*D*_0_/*D*_eff_)^1/2^ are observed, as substrate molecules preferentially interact with the walls. In conclusion, in our samples, the interaction of the substrate molecules with the pore walls clearly controls the transport, and this transport is additionally hindered in the LiChrospher Si 60 samples because of the small pore sizes, magnifying the effect of wall interactions.

### Evaluation of Mass Transfer Effects

Having scrutinized the diffusion of the relevant molecules within the particles now allows for addressing the question if the catalytic properties are indeed affected by mass-transfer limitations. In this respect, the dimensionless Weisz–Prater criterion Φ_WP_ (eq 3) was introduced to evaluate if mass transfer limits a reaction.^41^ In essence, it relates the reaction rate to effective diffusion in the pore space.^38^ The investigated DMAP-catalyzed acetylation reaction of alcohols has a reaction order of two, which defines that transport limitations would result in Φ_WP_ values above 0.3, according to the underlying concept.^39,40,63^ Although acetic anhydride, being one of the two reactants, is used in excess, which reduces the experimental reaction order to one, we enter a reaction order of two, based on the reaction mechanism.
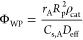
3where *r*_A_ is the observed reaction rate per catalyst mass, ρ_cat_ the density of the catalyst, *R*_p_ the particle radius (i.e., here 2.5 μm), *c*_s,A_ the surface concentration of the observed molecule, and *D*_eff_ the effective diffusivity.^41^ Note that the value of the density, ρ_cat_ of the catalyst, may not be obvious in the present case. Taking into account the underlying theory of the Weisz–Prater criterion Φ_WP_, the organocatalyst layer is negligible and therefore the overall skeleton density of the silica was chosen for ρ_cat_, and the intrinsic porosity of the particles is taken into account in *D*_eff_.

**Figure 12:**
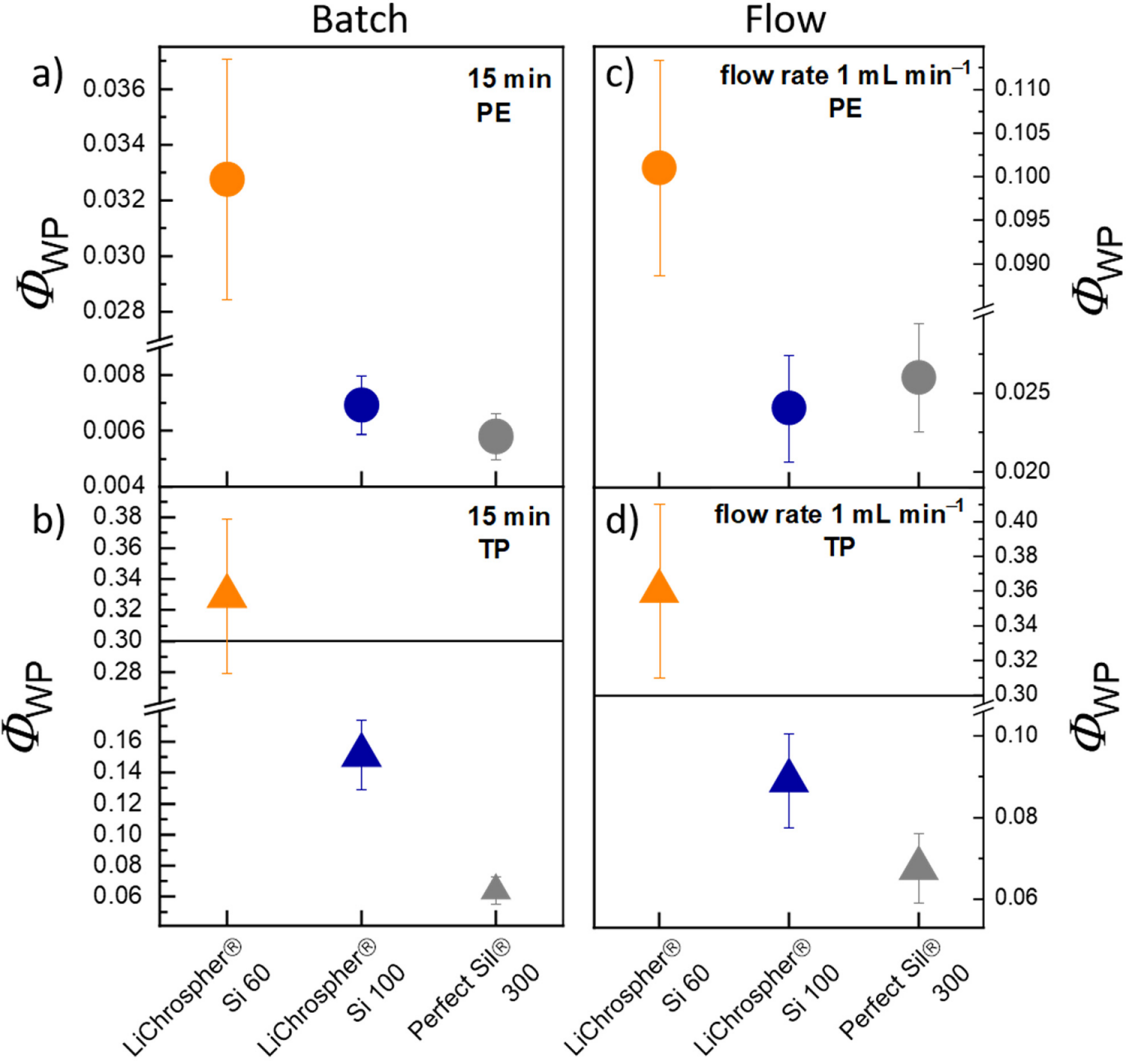
Values for the Weisz–Prater criterion Φ_WP_ calculated for batch **a-b** and flow catalysis **c-d** of 1-phenylethanol (**PE**) **a,c** and α-tocopherol (**TP**) **b,d**, respectively, employing the experimentally determined diffusion coefficients. A Φ_WP_ value above 0.3 correlates with a diffusion-controlled reaction.^39,40,63^

Figure 12a, b shows Φ_WP_ values for the batch catalysis, which were calculated from the initial reaction rate after 15 min, while for the flow experiments the catalytic data from the highest flow rate were chosen, in which mass transport limitations have the greatest impact due to short contact time between reagents and the surface (Figure 12c, d). The Φ_WP_ values were calculated with the Equations (S11) to (S15), based on the data shown in Table S6–S12, and depicted in Figure S18–19. In the case of **PE** (Figure 12a, c), all of the Φ_WP_ values, i.e., for all three mesopore diameters and in batch as well as flow mode, are rather small, indicating the absence of transport limitations. These findings were in agreement with Yadav et al., who showed that there were no internal diffusion limitations for the etherification of glycerol and **PE**, which was catalyzed by heteropolyacids immobilized on hexagonal mesoporous silica.^64^ The investigation of the hydrogenation of acetophenone to PE on a zeolithe- and MCM41 catalyst also demonstrates that the mass transfer rate is distinctly higher than the reaction rate.^65,66^

By contrast, the Φ_WP_ values for **TP** (Figure 12b, d) were significantly higher than **PE** due to the slower diffusion of the much larger molecule, as derived from the NMR studies. However, for the 6 nm particles, the calculated Φ_WP_ values for **TP** are larger than 0.3, which suggests that the reaction is heavily influenced by transport limitations. On the contrary, the small Φ_WP_ values calculated for the 10 and 30 nm mesopore particles indicate that the catalytic performance is not significantly determined by mass transfer limitations, being in agreement with the qualitative findings in batch and flow catalysis. Since there are no other studies on reactions with **TP**, we compared our calculated data with the esterification of different fatty acids with different alcohols like octanol^67^ or methanol.^68−70^ Here, the immobilization of the catalyst on Ion Exchange Resins,^67,68^ zeolites,^69^ and mesoporous SBA15^70^ catalysts resulted in no transport limitation for the respective reaction. However, it must be taken into account that in all these studies the diffusion coefficients were just estimated based on porosity *ε* and tortuosity τ. Such estimates of the effective diffusion coefficient *D*_eff_ are highly error-prone and therefore distort the calculated Φ_WP_ values. In the present work, we have determined *D*_eff_ experimentally from diffusion experiments and appropriate models, yielding more meaningful values.

We conclude that the 10 and 30 nm particles therefore are a suitable carrier material for liquid-based organocatalysis and also for larger reactant molecules such as **TP**. The work of Sun et al. shows identical limitations for the hydrotreatment of gaseous hexadecane over a Pt and HPMo (phosphomolybdic acid) catalyst on SBA-15 with 5, 7, and 10 nm mesopores. Here, the product conversion increases with the mesopore diameter as well and only the 10 nm porous material possesses Φ_WP_ values smaller than the corresponding limit and hence, the absence of diffusion limitations.^71^ Although in Sun’s study, the reaction was performed in the gas phase, interestingly a similar optimum mesopore dimension (10 nm) was found, being in agreement with our study.

## Conclusion

We addressed the fundamental question of whether heterogeneous organocatalysis in mesoporous scaffolds is significantly affected by mass transport limitations, using a well-known organocatalyst (DMAP) immobilized in mesoporous silica and performing the reactions in liquid medium. Three types of mesoporous particles of the LiCrosphere-series were selected, being commercially utilized in liquid chromatography, which possess mesopores of 6, 10, and 30 nm in diameter, respectively, and which are optimized for high permeability. In comparison to previous studies addressing the impact of the mesoporosity, one major feature of the present study is the thorough experimental determination of the intraparticular self-diffusion constant of the reactant molecules (1-phenylethanol **PE** and α-tocopherol **TP**) and acetylated molecules by the PFG NMR method, rather than using semiquantitative estimations for this quantity. Notably, a specially designed experimental procedure was established to ensure that the PFG NMR experiment provides the self-diffusion coefficient within the mesoporous particles only, i.e., excluding contributions from diffusion within the interparticular space.

As exemplified by the acylation reaction of **PE** and **TP**, the 6 nm-mesopore material possesses the largest surface area but does not constitute an efficient carrier material for catalytic applications. The mesoporous network small connecting pores (<3 nm) results in distinct pore blocking effects, as revealed by physisorption, as well as strong liquid-surface interactions, suggested by PFG NMR. The two features lead to restricted transport inside the mesopore space and thus poor catalytic performance especially for larger molecules such as α-tocopherol **TP**. However, both other materials (10 and 30 nm mesopores) possess a more open mesoporous network with distinctly larger connecting pores that enable comparably advanced mass transport of reactants to the catalyst on the surface and therefore a higher catalytic performance, despite a lower internal interface. The observed yields and TOF values suggest that these two types of mesoporous materials are potentially suitable carrier materials for heterogeneous organocatalysis.

Furthermore, we calculated the so-called Weisz–Prater-Criterion Φ_WP_ by using experimental values (PFG NMR) for the self-diffusion coefficient. The concept of Φ_WP_ originally is a macroscopic approach to identify a possible impact of mass transport limitations on a catalyzed reaction, which is therefore often applied to porous catalyst media. While the interpretation and application of the dimensionless Φ_WP_ is a matter of discussion and perhaps even debatable, the determined Φ_WP_ are significantly different for the three materials and thus allow for a valid comparison. The Φ_WP_ values for the 6 nm material indicate substantial mass transport limitation, and the differences are already evident based on comparing the self-diffusion coefficients. Our work therefore supports the usability of the Weisz–Prater-Criterion, being mostly used for reactions in the gas phase and also in flow catalysis in solvent-based organocatalysis. Yet, our study demonstrates that the necessary parameters entering Φ_WP_ have to be carefully assessed, especially the diffusion constants.

We expected higher yields for the esterification of the sterically more demanding **TP** molecule using the functionalized 30 nm mesoporous particles compared to the particles possessing smaller mesopores. However, this trend is only visible under batch conditions, while the acetylation of **TP** under flow conditions results in conversions and TOF values similar to those for the 10 nm-pore material. Interestingly, the experimentally determined diffusion coefficients of the 10 and 30 nm materials differ only slightly, thus indicating similar diffusion behavior. Thus, on the basis of the diffusion coefficients there should not be a significant difference between the catalytic performance between batch and flow catalysis, if the 10 and 30 nm porous particles are compared. We believe that this interesting difference might be explained by flow conditions being favorable to overcome the observed liquid-surface interactions and also the trapping of substrates inside the mesoporous network. To better understand the underlying transport processes, we intend to characterize the flow properties of the packed flow-reactors with techniques commonly in separation science, like recording van Deemter-plots. A comparison with the flow catalysis data will eventually help to evaluate which flow conditions are most beneficial for the catalysis.

Finally, the choice of an optimal pore size for heterogeneous organocatalysis therefore depends on several factors, namely, reactant dimension, mesopore connectivity, solvent, and flow conditions. This study shows that a continuous flow setup is in general beneficial for immobilized catalysts—not only for easier workup and recycling, but also to use the full potential of the functionalized material. Also, our study provides a conceptual method for advancements in heterogeneous catalysis in flow in general, in that knowledge-driven progress needs a comprehensive choice of the materials (optimized for flow) and state-of-the-art characterization, including the determination of the self-diffusion coefficient in the respective materials and a thorough characterization of the mesopore space. Further advancements might include nanotomography on the porous materials to obtain a reconstructed digital mesopore space for which diffusion coefficients might be simulated and compared to the experimental values.

## Supporting Information Available

Detailed calculations of the catalyst loadings from the elemental analysis data and recorded DRIFT spectra. Additional characterization of larger mesopores was carried out by Mercury Intrusion Porosimetry. For further evaluation and distinction of pore-blocking-effects and cavitation Ar physisorption measurements, complete Hysteresis Scanning isotherms, and the corresponding fitting curves of the NLDFT kernel were depicted in the Supporting Information. A detailed physisorption analysis of the 5 and 10 μm silica particles, which were used for the verification of the obtained diffusion coefficients is also included. To complement the PFG-NMR diffusion experiments additional information about the sample preparation and its effects on diffusion attenuation curves, the determined diffusion coefficients and factors influencing the echo decay shapes. The detailed calculation and the used data as well as a tabular values of Φ_WP_ for flow- and batch conditions (PDF)
