# Supplementary material for: Influence of the Mesoporosity of Silica Carrier Materials on the Performance of an Immobilized Organocatalyst in Heterogeneous Catalysis
Source: ACS Appl Mater Interfaces. 2025 Apr 10;17(16):24283–99. doi: 10.1021/acsami.4c19398 (PMC12022970; doi:10.1021/acsami.4c19398)
Supplement: Supplementary file 1 — am4c19398_si_001.pdf [file am4c19398_si_001.pdf]

# Supporting Information

# Influence of the Mesoporosity of Silica Carrier Materials on the Performance of an Immobilized Organocatalyst in Heterogeneous Catalysis

*Aline Trommer[a], Janis Hessling[b], Peter R. Schreiner[c,d], Monika Schönhoff[b]*,*

*and Bernd M. Smarsly[a,d]**

[a]Institute of Physical Chemistry, Justus-Liebig-University, D-35392 Giessen, Germany

[b]Institute of Physical Chemistry and Center of Soft Nanoscience, Westfälische Wilhelms-University, D-48148 Münster, Germany

[c]Institute of Organic Chemistry, Justus-Liebig-University, D-35392 Giessen, Germany

[d]Center for Materials Research, Justus-Liebig-University, D-35392 Giessen, Germany

Electronic Supporting Information

# Table of contents

Synthesis and characterization of precursors ........ 3
(3-azidopropyl)trimethoxysilane ........ 3
DMAP click-derivate ........ 3
Functionalization and calculation of catalyst loading ........ 4
Diffuse reflectance infrared fourier transform spectroscopy (DRIFT) ........ 5
Mercury Intrusion Porosimetry ........ 6
Physisorption ........ 6
Isotherm and PSD of 5 µm and 10 µm sized silica particles ........ 6
$N_2$ physisorption isotherms of unfunctionalized and azide-functionalized materials . 7
Fitting error for NLDFT analysis ........ 8
Distinction of Cavitation and Pore-blocking-effect ........ 9
Complete hysteresis scanning isotherms ........ 11
PFG-NMR Diffusion ........ 12
Sample preparation ........ 12
Effect of sample drying on diffusion attenuation curves ........ 12
Diffusion of molecules in bulk solution ........ 13
Diffusion in porous particles: Factors influencing the echo decay shape ........ 13
(i) Distribution of diffusion coefficients ........ 13
(ii) Internal magnetic field gradients ........ 14
(iii) Restricted diffusion in a sphere according to the model of Balinov et al. ........ 17
Calculation of $\Phi_{WP}$ and corresponding data ........ 20
Calculation of $r_a$ (observed reaction rate per catalyst mass) ........ 20
Calculation of $\Phi_{WP}$ ........ 21
Complete $\Phi_{WP}$ values for batch and flow catalysis ........ 22
References ........ 23

## Synthesis and characterization of precursors

The synthesis of (3-azidopropyl)trimethoxysilane and DMAP click-derivative was done according a literature described synthesis route.[1,2]

### (3-azidopropyl)trimethoxysilane

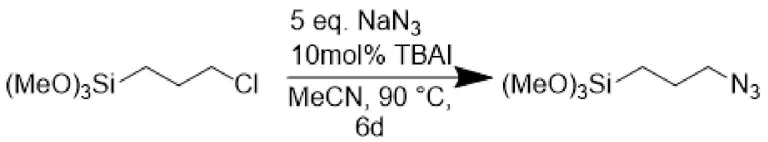


***Figure S1.*** *Reaction scheme for azide-linker synthesis*

1.763 g sodium azide (27.12 mmol) and 0.202 g tetrabutylammonium iodide (0.55 mmol) were added to a Schlenk flask and dried under vacuum for 2 h. Under an inert gas atmosphere, the solids were dissolved in 6.5 mL dry acetonitrile and 1 mL chloroproyl-trimethoxysilane (5.42 mmol) were added. The solution was heated to 90°C under reflux conditions for 6 d. The resulting precipitate was filtered off, rinsed with 5 mL of dry acetonitrile and the solvent was removed under reduced pressure. The crude product was dissolved in 5 mL dry pentane and stored overnight at -20°C. The solid residues were filtered off, rinsed with 5 mL dry pentane and the solvent was again removed under reduced pressure. The final product was a colourless liquid, which was analysed by $^1$H-NMR.

$^1$H-NMR (400 MHz, $CDCl_3$, 298 K): $\delta$ (ppm) = 3.51 (s, 9H), 3.20 (t, 2H), 1.64 (p, 2H), 0.63 (t, 2H).

### DMAP click-derivate

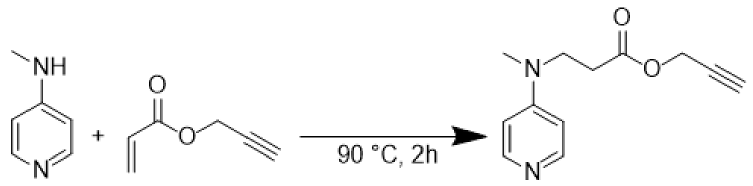


***Figure S2.*** *Reaction scheme for the alkin-functionalized 4-methylaminopyridine derivate.*

In a round-bottom flask 0.250 g 4-methylaminopyridine (2.36 mmol) was dissolved in 1 mL propargyl acrylate (9.04 mmol) and heated for 2 h at 90°C under reflux. To remove excess propargyl acrylate, the solution was distilled under vacuum (10 mbar) at 90°C and the remaining orange liquid was purified by column chromatography (silica gel DCM:MeOH 10:1, 1 % TEA). 0.506 g (98.3 %) of a white-brownish solid was isolated. The final product was analyzed by HRMS (ESI-TOF).

HRMS (ESI-TOF): $[M+H]^+$ calculated for $C_{12}H_{14}N_2O_2$: m/z=219.1128; we found m/z=219.1131

## Functionalization and calculation of catalyst loading

The azide loading of the functionalized materials was calculated with the elemental analysis data (**Table S1**) as follows[1]:

$$X_{\text{azide}} = \frac{\%N}{N(\text{N})M(\text{N})}, \quad \text{(S1)}$$

$X_{\text{azide}}$ azide loading (mmol g$^{-1}$)

$\%N$ nitrogen content

$N$(N) number of nitrogen atoms in azide (3)

$M$(N) molar mass of nitrogen

The DMAP loading of the silica materials was calculated as follows[1]:

$$\%N_{DMAP} = \frac{\%N_{EA}(100-(\%N_{azide}+\%C_{azide}+\%H_{azide}))}{100-(\%N_{EA}+\%C_{EA}+\%H_{EA})}, \quad \text{(S2)}$$

$$X_{DMAP} = \frac{\%N_{\text{DMAP}}-\%N_{\text{azide}}}{N_{\text{DMAP}}(\text{N})M(\text{N})}, \quad \text{(S3)}$$

$X_{\text{DMAP}}$ DMAP loading (mmol g$^{-1}$)

$N_{\text{DMAP}}$(N) number of additional nitrogen atoms from second functionalization step (= 5)

***Table S1:*** *Elemental analysis data and corresponding loadings*

| | LiChrospher® Si60 5 µm | LiChrospher® Si100 5 µm | Perfect Sil® 300 5 µm |
|---|---|---|---|
| $N\%_{(\text{Azide})}$ / % | 1.965±0.006 | 1.210±0.004 | 1.350±0.004 |
| Azide loading / mmol g$^{-1}$ | 0.47±0.14 | 0.29±0.09 | 0.32±0.10 |
| $N\%_{(\text{DMAP}))}$ / % | 1.680±0.005 | 1.810±0.005 | 1.275±0.004 |
| DMAP loading / mmol g$^{-1}$ | 0.26±0.07 | 0.26±0.08 | 0.18±0.05 |

## Diffuse reflectance infrared fourier transform spectroscopy (DRIFT)

The introduced azide linker shows a characteristic IR-band around 2150 $cm^{-1}$. Upon the following click reaction with the DMAP derivate, the intensity of this band decreases. The DRIFT spectra were recorded by a Bruker alpha in a range of 400-4000 $cm^{-1}$ and a resolution of 2 $cm^{-1}$.[1]

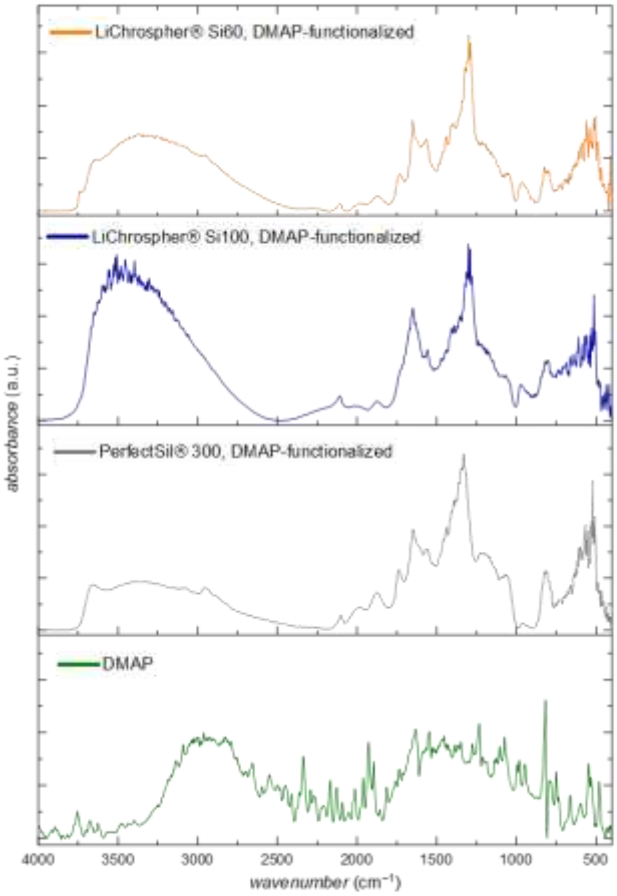


***Figure S3***. *Full spectrum and zoom of the DRIFT spectra of the azide- and DMAP-functionalized materials.*

# Mercury Intrusion Porosimetry

Physisorption is the method of choice for mesoporous materials (diameter between 2-50 nm). Since the PerfectSil® 300 particles possess a pore-size distribution (PSD) with pores greater than 50 nm, we performed MIP measurements to verify the physisorption based PSD.[3]

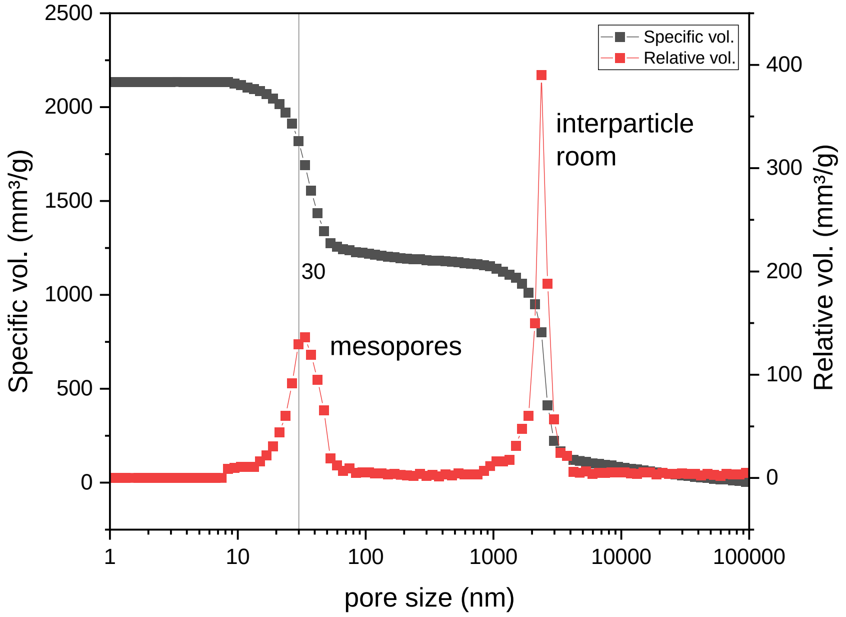


***Figure S4***. *MIP measurement for characterization of the mesoporosity of the PerfectSil® 300 particles.*

# Physisorption

## Isotherm and PSD of 5 µm and 10 µm sized silica particles

For a verification of the diffusion model employed, LiChrospher® Si100 particles with 10 µm diameter were used. Physisorption measurements show that the LiChrospher® Si100 5 µm and 10 µm have a similar mesoporous structure.

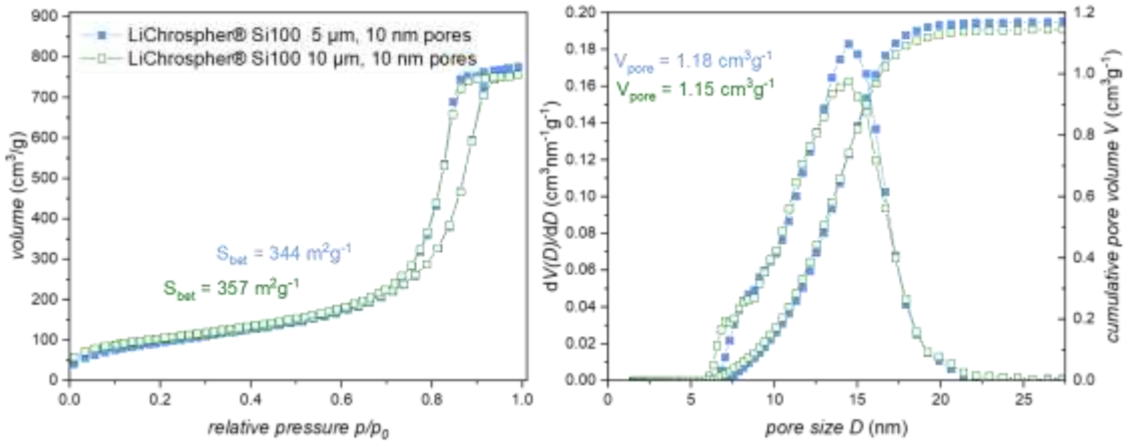


***Figure S5***. *Physisorption isotherms and differential and cumulative PSD ($N_2$, 77 K, NLDFT for zeolites/silica, cylindrical pores, adsorption branch, MPA of 5) for 5 µm and 10 µm LiChrospher® Si100 particles.*

## $N_2$ physisorption isotherms of unfunctionalized and azide-functionalized materials

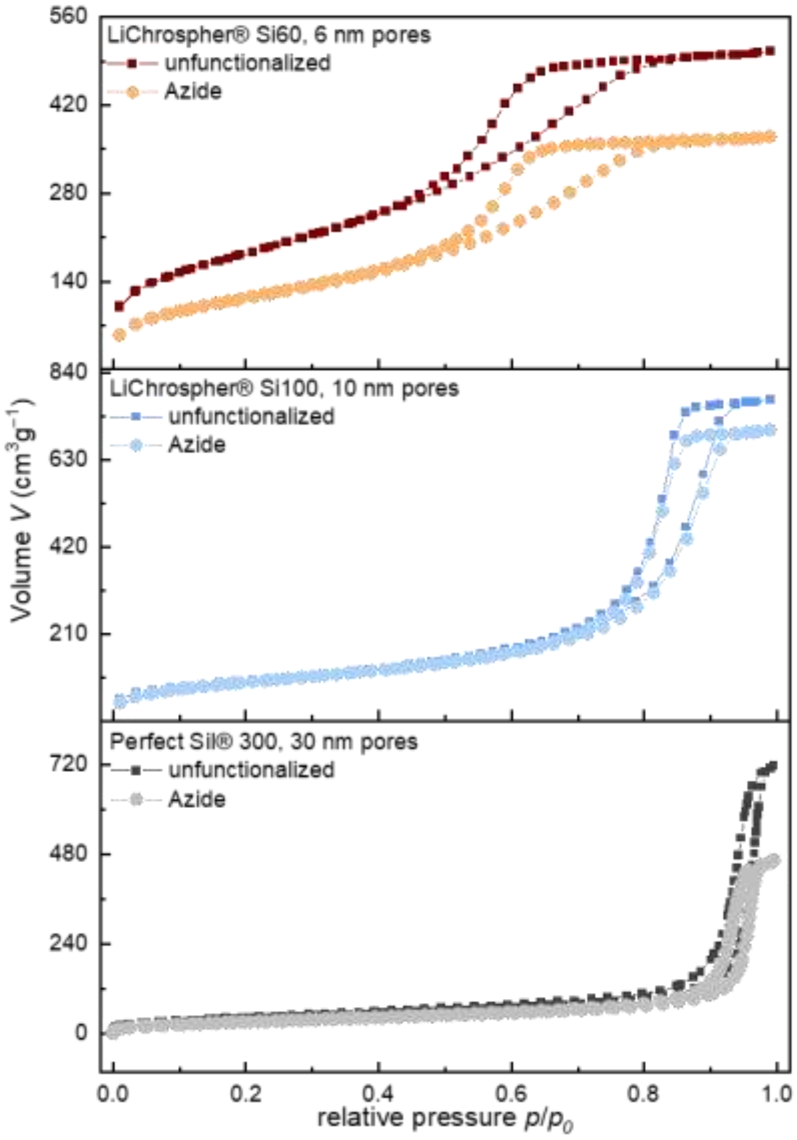


***Figure S6.*** *$N_2$-physisorption isotherms of the unfunctionalized and azide-functionalized materials.*

## Fitting error for NLDFT analysis

Since the fitted curve of the NLDFT analysis does not depict low relative pressure values correctly, the PSD of small meso- and micropores should be evaluated with precautions, especially for the functionalized materials.

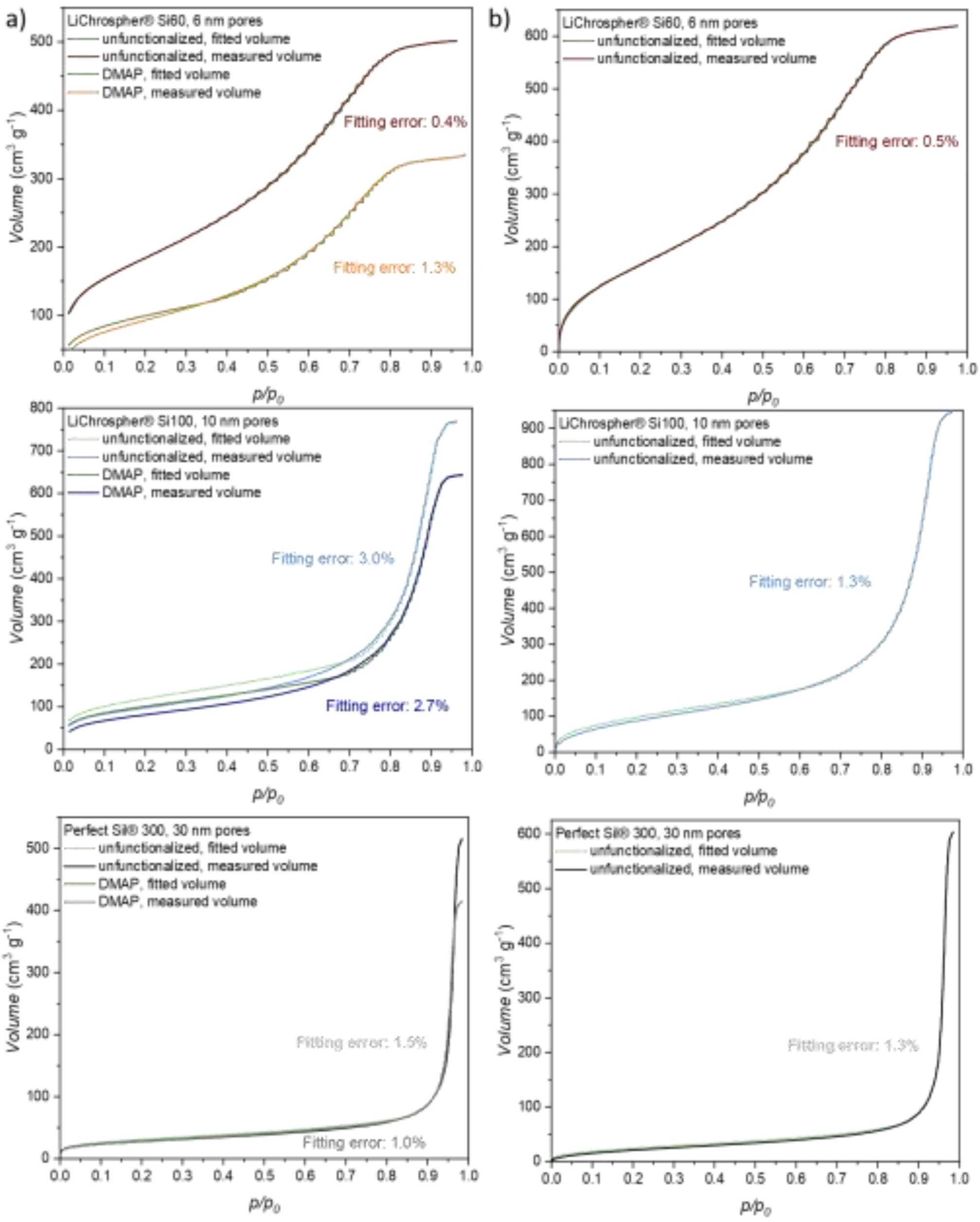


***Figure S7. a*** *NLDFT fitting quality (i.e. experimental data and fitting curve) of the $N_2$ physisorption isotherms for the unfunctionalized and functionalized materials,* ***b*** *NLDFT fitting quality for the Ar physisorption isotherms for the unfunctionalized materials.*

## Distinction of Cavitation and Pore-blocking-effect

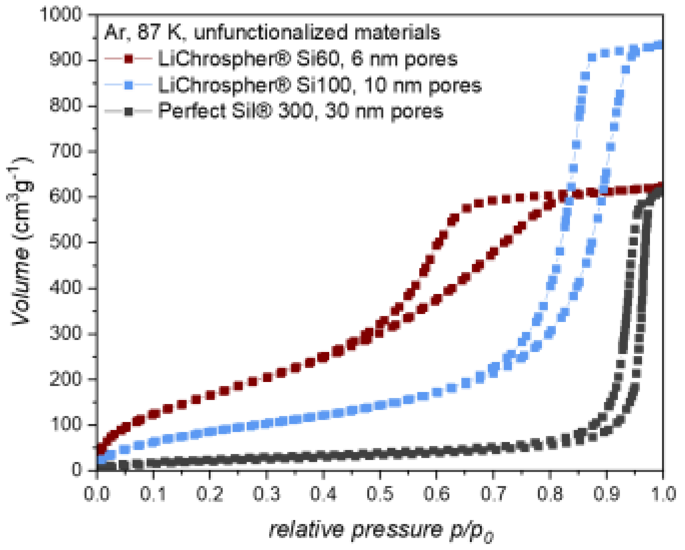


*Figure S 8*

***Figure S8**. Ar-isotherms of the unfunctionalized silica materials (Ar 87 K, NLDFT for zeolites/silica, cylindrical pores, MPA of 5).*

To distinguish pore-blocking from cavitation effects, a variation of the adsorptive is necessary, because the critical pore width depends on the chemical nature of the adsorptive. The measured Ar- isotherms are shown in **Figure S** 8. Since all materials show similar PSD with either $N_2$ or Ar, the observed differences in adsorption- and desorption-based PSD are due to pore-blocking effects and not due to cavitation phenomena (**Figure S** 9).[4]

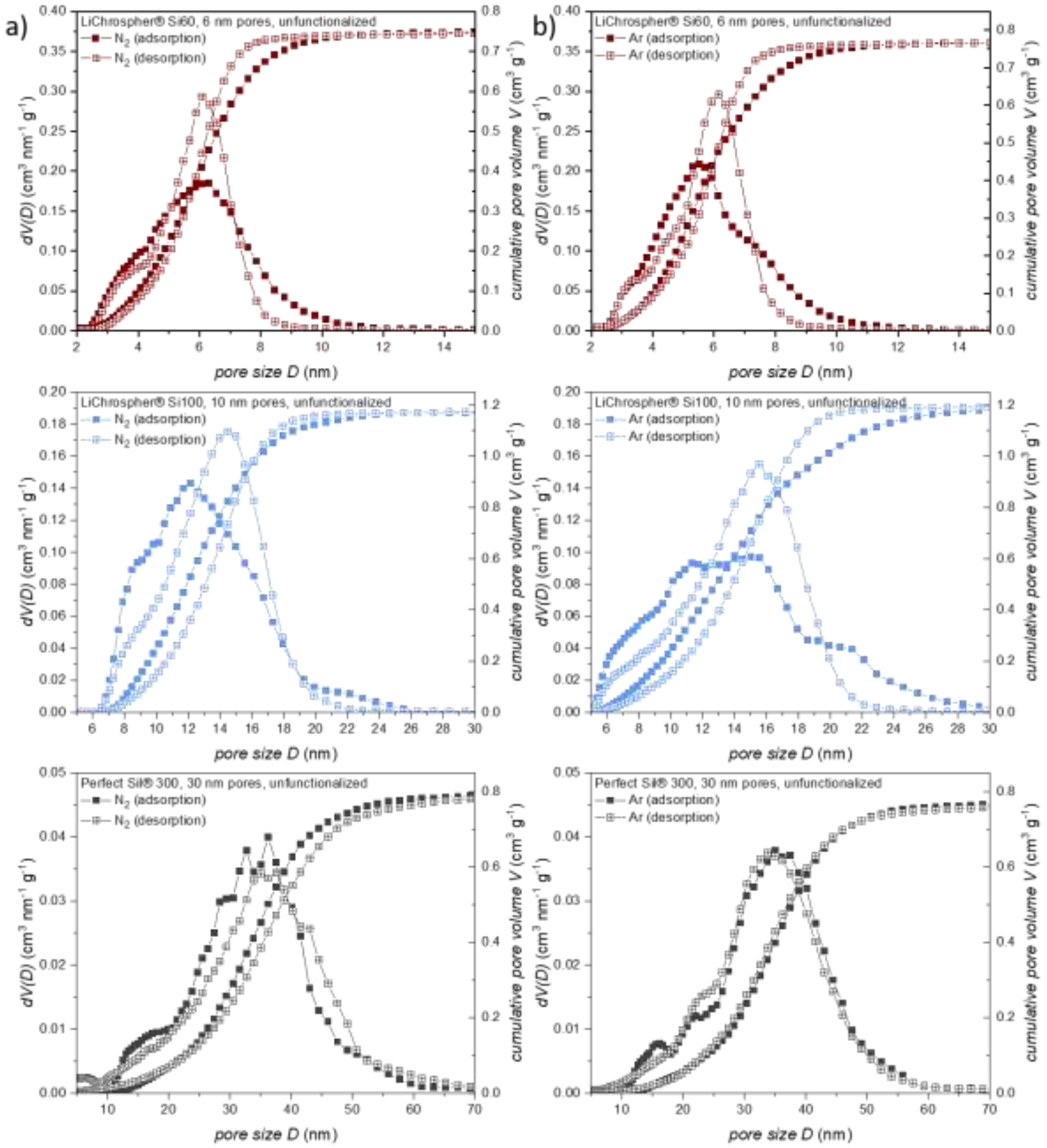


*Figure S 9*

***Figure S9**. Comparison of $N_2$ (**a**) and Ar-PSD (**b**) proves that the delayed desorption is caused by the pore-blocking effect, as the shape of the PSD are very similar.*

## Complete hysteresis scanning isotherms

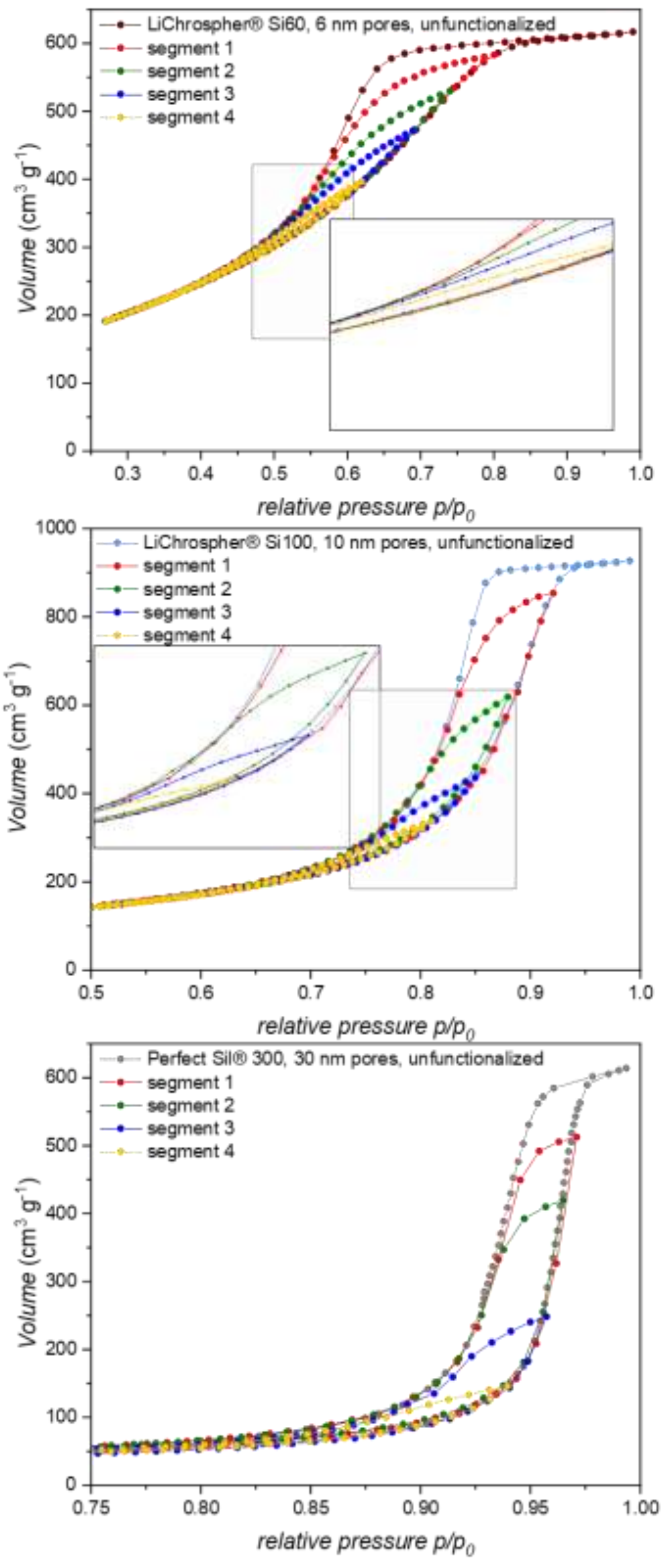


***Figure S10.*** *Hysteresis scanning isotherms with both adsorption as well as desorption isotherms depicted (using Ar, 87 K).*

## PFG-NMR Diffusion

### Sample preparation

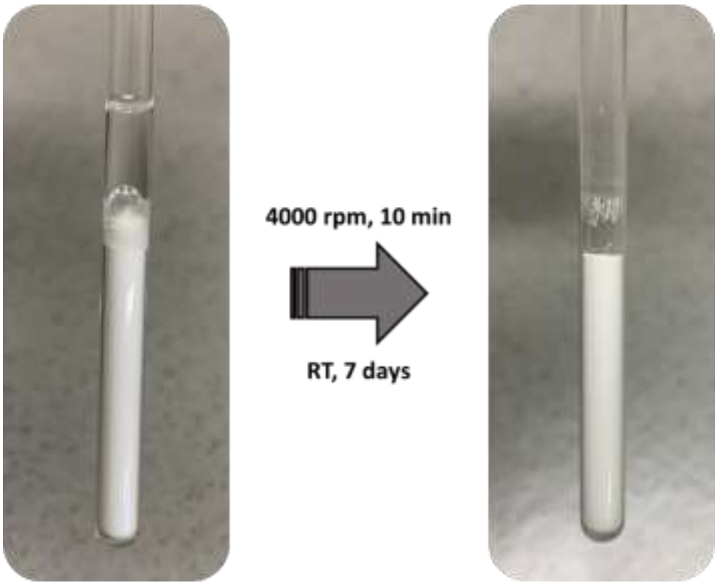


***Figure S11***. *Sample before centrifugation and after the 7-d waiting period, employing an amount of solution which allows complete pore filling without excess solution in the interstitial space.*

### Effect of sample drying on diffusion attenuation curves

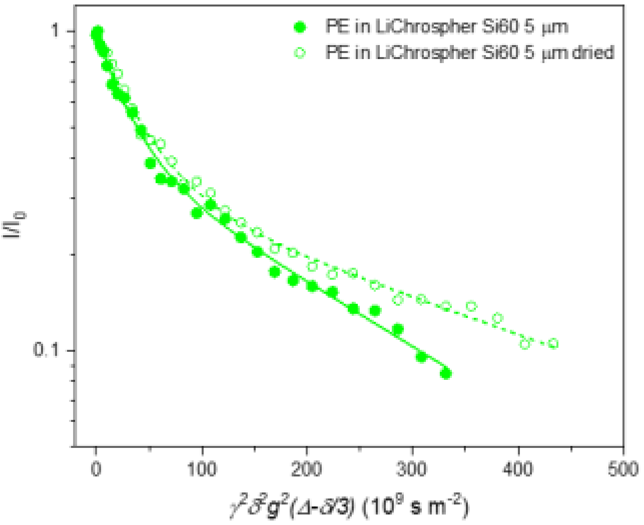


***Figure S12***. *Diffusion attenuation curve of phenylethanol in LiChrospher® Si60, dried (empty symbols) and undried (filled symbols) with a biexponential fit.*

## Diffusion of molecules in bulk solution

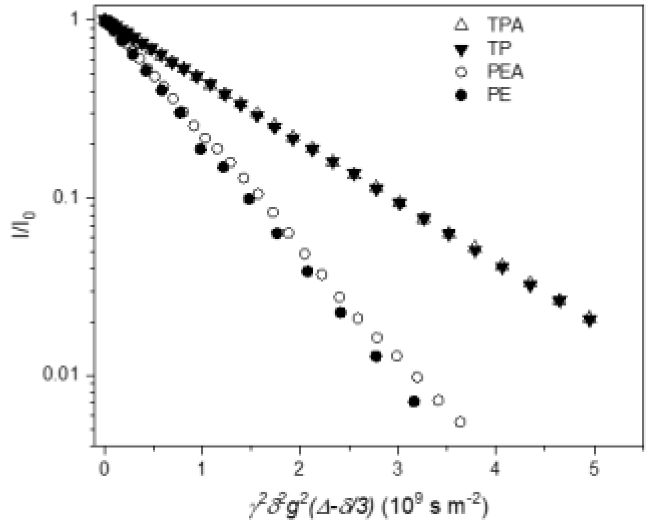


***Figure S13**. Diffusion attenuation curves of phenylethanol **PE** (filled circles), phenylethylacetate **PEA** (empty circles), tocopherol **TP** (filled triangles), and tocopherolacetate **TPA** (empty triangles) in bulk liquid.*

***Table S2**. Diffusion coefficients of the molecules in solution and hydrodynamic diameters of the molecules derived from their respective diffusion coefficients and the Stokes-Einstein equation (**Equation S4**)*

| Bulk | phenylethylacetate | phenylethanol | tocopherolacetate | tocopherol |
|---|---|---|---|---|
| $D$ / m$^2$ s$^{-1}$ | (1.48±0.01)x10$^{-9}$ | (1.57±0.01)x10$^{-9}$ | (7.88±0.02)x10$^{-10}$ | (7.91±0.03)x10$^{-10}$ |
| $d_h$ / nm | 0.55 ± 0.03 | 0.52±0.03 | 1.03±0.05 | 1.02±0.05 |

$$d_\mathrm{h} = \frac{kT}{3\pi\eta D} \qquad (S4)$$

## Diffusion in porous particles: Factors influencing the echo decay shape

### (i) Distribution of diffusion coefficients

In a porous material with a heterogenous pore size distribution or heterogenous pore geometries a distribution of diffusion coefficients may exist[5], which is, however, not necessarily detectable by PFG-NMR. Heterogeneous pore sizes only lead to nonexponential echo decays if the exchange time of the molecules between regions with different diffusivity, $\tau_{ex}$, is on the order of or larger than the observation time $\Delta$ of the experiment. If the exchange is sufficiently slow, i.e., $\tau_{ex} >> \Delta$, no averaging during the observation time occurs and a multiexponential echo-decay can be observed. If the exchange is fast, i.e., $\tau_{ex} << \Delta$, an exponential echo decay is observed with an average diffusion coefficient of the species.

The silicas used in this study have a known heterogeneous pore size distribution and in addition 'bottle-necks' were detectable through physisorption in LiChrospher® Si60 + Si100. The effect these 'bottle-necks' can have on the exchange between different species and the echo decay curve is illustrated in **Figure S14**.

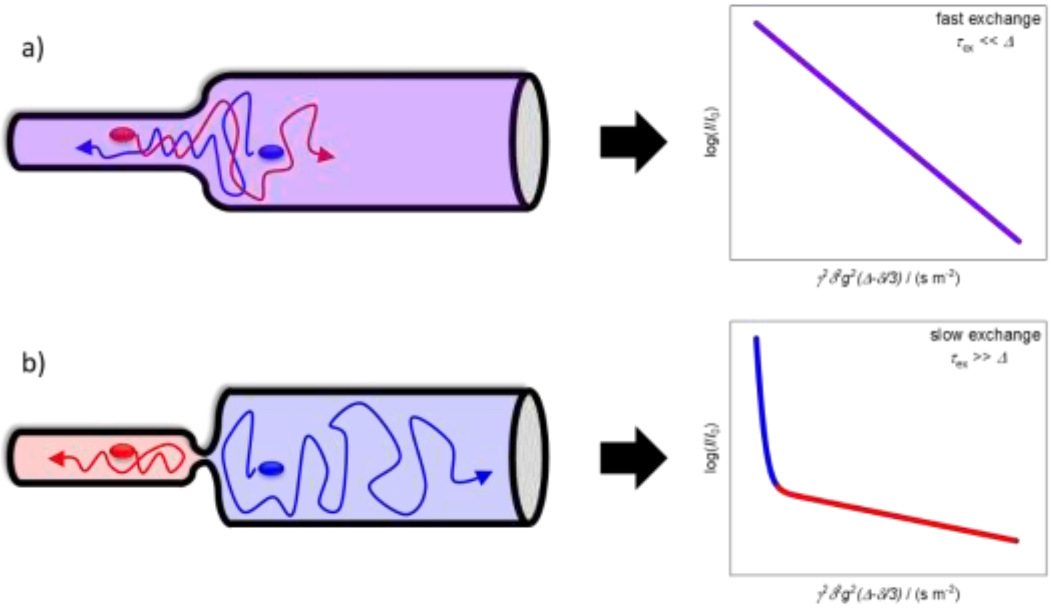


***Figure S14.*** *Sketch of the diffusion pathway of two molecules in two different pore size regions and the effect on the echo decay curve, where (a) the exchange between the molecules is unhindered and (b) the exchange is hindered by a bottle neck.*

**Figure S14** shows a simplified sketch of the diffusion pathway of two molecules in a region with different pore sizes. In the first case (a) the exchange between the two regions is unhindered. Both molecules experience both pore regions and therefore yield identical rms displacements during the observation time $\Delta$. This is the case of fast exchange ($\tau_{ex} << \Delta$), where an exponential echo decay with only one averaged diffusion coefficient is observed. In the second case (b) the exchange between the two regions is hindered by a 'bottle-neck' ($\tau_{ex} >> \Delta$). Each molecule experiences just one pore region during the observation time $\Delta$, resulting in different rms displacements and different slopes of the echo decay. Finally, this results in a superposition, i.e., a biexponential echo decay with a steeper slope for the faster molecule.

To estimate whether such 'bottle-necks' can be the sole reason for the non-exponentiality of the echo decays in **Figure 8**, we can estimate the mean square displacement of the slower species. For a 3-dimensional diffusion it can be calculated according to $\langle R \rangle^2 = 6D\Delta$. With the value of $\Delta$ of 6 ms and values for the slower species of $D$ of around $5 \times 10^{-12}$ $m^2 s^{-1}$, the estimated root mean square displacement would be around 400 nm. However, not in all silicas 'bottle-necks' are detectable (see Perfect Sil® 300) and most importantly, a mesoscopic heterogeneity with distinguishable regions on the order of 400 nm with deviating pore size might be unlikely in particles with radii of 2.5 µm or 5 µm. We conclude that heterogeneous pore size distributions can not fully explain the deviation from an exponential decay as seen in **Figure 8**.

## (ii) Internal magnetic field gradients

In NMR diffusion experiments artefacts arising from internal magnetic field gradients are a common occurrence.[6–11] In a diffusion experiment two pulses of a magnetic field gradient with a magnitude of $g$ (**Figure S15**) are used to track the displacements of the spins. The first gradient pulse labels the position of the spins by dephasing the magnetization. If there is no displacement of the spin, the second gradient refocusses the magnetization completely. If the spin are displaced by diffusion during the observation time $\Delta$ between the gradients, the refocussing is not complete, which causes an attenuation of the spin-echo signal. Here, a stronger magnitude of the gradient ($g$) causes a higher attenuation of the signal and thus a variation of $g$ leads to the typical echo attenuation curve (**Figures**

**8, Figure S12** and **Figure S13**). In case of an exponential decay the diffusion coefficient $D$ can be derived by the Stejskal-Tanner equation (**Equation 1**).

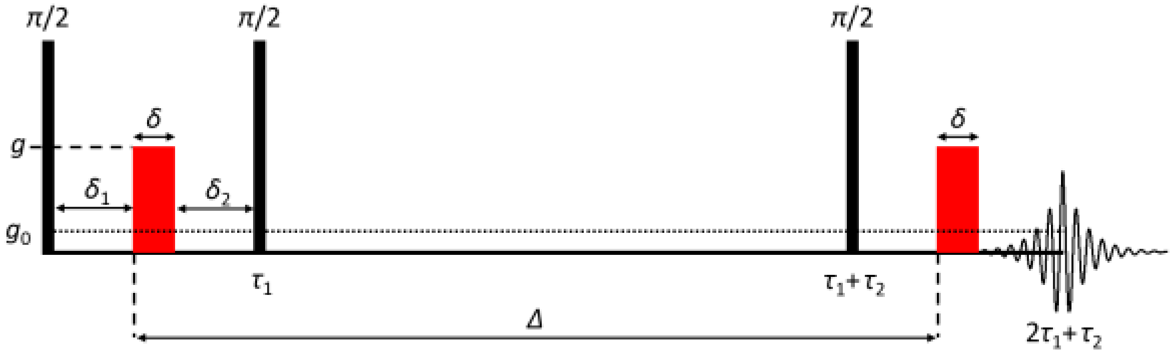


***Figure S15*** *The stimulated echo pulse sequence with magnetic field gradient pulses in red and rf pulses in black.*

Ideally, only the deliberately applied pulsed magnetic gradients should be present during the diffusion experiment. However, often internal, static magnetic field gradients exist (magnitude of $g_0$), which cause unwanted dephasing and rephasing of the spins and subsequently alter the diffusion attenuation curves. The attenuation curves in the case of background gradients can be described by **Equation S5**[8], where $\tau_1$, $\tau_2$, $\delta_1$ and $\delta_2$ are delay times, see **Figure S15**. The signal attenuation has contributions from the deliberately applied pulsed magnetic gradients ($g$ term), the unwanted internal magnetic field gradients ($g_0$ term) and a cross-term from both magnetic gradients ($gg_0$ cross-term).

$$\ln[E(\delta,\Delta,g)] = -\gamma^2 D \left\{ \overbrace{g^2\delta^2\left(\Delta - \frac{\delta}{3}\right)}^{g\text{ term}} + \overbrace{g_0^2\tau_1^2\left(\tau_2 + \frac{2}{3}\tau_1\right)}^{g_0\text{ term}} \right.$$

$$\left. + \underbrace{gg_0\delta\left[2\tau_1\tau_2 + 2\tau_1^2 - \frac{2}{3}\delta^2 - \delta(\delta_1+\delta_2) - (\delta_1^2+\delta_2^2)\right]}_{gg_0\text{ cross-term}} \right\} \tag{S5}$$

The influence of the $g_0$ term on the diffusion attenuation can be neglected, because it is constant and has no effect on the slope of the $g$-dependent attenuation curve. However, the cross-term is not constant and does affect the slope of the attenuation curve. Especially for large values of $g$ the cross-term may become significant, this could lead to deviations from the exponential decay as those seen in **Figures 8**, **Figure S12** and **Figure S15**.

In porous media internal magnetic field gradients can arise from magnetic field inhomogeneities within the individual pores or the diamagnetic magnetization of neighboring particles.[7] To determine if the cross-term is significant in the present material the magnitude of the internal gradient needs to be estimated. This can be done by following a method described in the literature, where the linewidth of the spectra of bulk liquid is compared to that of liquid confined in the porous material.[9] By observing the broadening in the linewidth $\Delta\nu$, the magnetic susceptibility $\Delta\chi$ can be derived from[12]:

$$\Delta\nu = \frac{\gamma\Delta\chi B_0}{2\pi} \tag{S6}$$

The internal magnetic field gradients can then be estimated with[10],

$$g_0 \approx \left(\frac{\gamma}{D}\right)^{\frac{1}{2}} (\Delta\chi B_0)^{\frac{3}{2}} \tag{S7}$$

where $D$ is the diffusion coefficient of the confined liquid.

The linewidths for each compound have been extracted from $^1$H spectra for each porous material, employing the $CH_3$-group of phenylethanol, the $CH_3$-group closest to the phenyl unit of phenylethylacetate and the terminal $CH_3$-groups of the alkyl chain of tocopherol and tocopherolacetate, yielding a significant broadening in comparison to the bulk liquid, see **Table S3**. From averaged linewidths the magnetic field gradients for each silica type are calculated according to **Equation** (**S6**) and (**S7**), and shown in **Table S3**.

***Table S3.*** *Linewidth of the $CH_3$-group of each sample and the estimated internal magnetic field gradient $g_0$ as an average for the respective silica*

| | Bulk liquid | Perfect Sil® 300 5 µm | LiChrospher® Si100 5 µm | LiChrospher® Si60 5 µm | LiChrospher® Si100 10 µm |
|---|---|---|---|---|---|
| $\Delta\nu$(PEA) / Hz | 2.70 | 381 | 436 | 450 | 457 |
| $\Delta\nu$(PE) / Hz | 4.46 | 350 | 436 | 462 | 419 |
| $\Delta\nu$(TocA) / Hz | 5.15 | 358 | 322 | 370 | 338 |
| $\Delta\nu$(Toc) / Hz | 3.90 | 347 | 338 | 421 | 318 |
| $g_0$ / T m$^{-1}$ | | 2.1 | 2.7 | 5.3 | 2.3 |

These internal magnetic field gradients are small compared to the applied pulsed magnetic gradient, which amounts to $g$ up to 25 T m$^{-1}$, but they are not negligible. Therefore, the deviation of the diffusion attenuation curves from an exponential decay can at least partially be explained by the existence of background gradients.

In order to monitor the influence of the internal gradients, we performed exemplary diffusion experiments on phenylethanol in LiChrosper® Si100 with different values of $\Delta$, which causes a variation of the dominant term $2\tau_1\tau_2$ in the see brackets of the cross-term (see **Equation S5**). As can be seen in **Figure S16**, the deviation from the initial slope at higher $g$ values become more pronounced for longer observation time $\Delta$, whereas the initial slope (down to $I/I_0$ = 0.6) stays almost the same. A similar behavior, though with a less pronounced nonexponential decay, was also found in the literature for the diffusion of different liquids in controlled pore glasses (CPG).[7] There the nonexponential behavior was explained by internal gradients over the length scale of the CPG grains, caused by diamagnetic magnetization of the neighboring particles. The initial decay showed no dependence on $\Delta$ and was therefore used to obtain the diffusion coefficient because the effect of the background gradient was deemed negligible in this region of the attenuation curve.[7] Similarly, we also fitted just the initial decay, due to it being less affected by the internal magnetic field gradients, especially at low diffusion times ($\Delta$ = 6 ms).

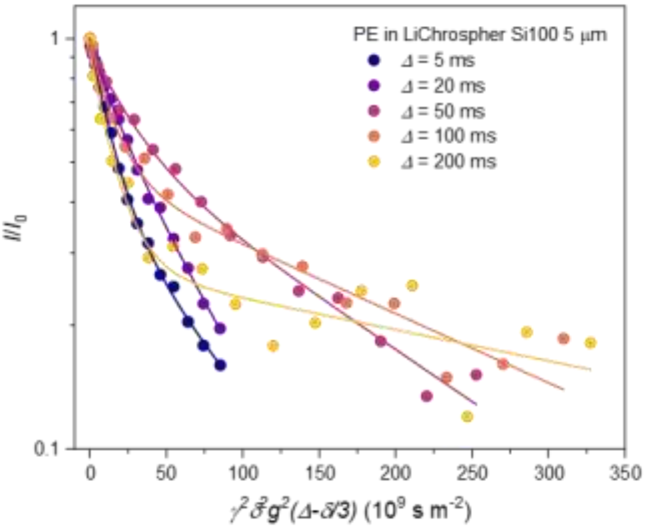


***Figure S16*** *Diffusion attenuation curve of phenylethanol in LiChrosper® Si100 for different diffusion times Δ. The lines are guide to the eye to emphasize the nonexponential behavior.*

## (iii) Restricted diffusion in a sphere according to the model of Balinov et al.

In the case of a complex diffusion mechanism often approximations and limits are used to simplify equations. For the case of the short-gradient-pulse limit, where $\delta \rightarrow 0$, the spin-echo decay can be described by

$$E(\delta, \Delta, g) = \iint \rho(r_0) P(r_0|r, \Delta) \exp[i\gamma g \delta (r_0 - r)] dr dr_0 \tag{S8}$$

where $P(r_0|r, \Delta)$ is the probability to find a spin starting at the position $r_0$ in position *r* after a diffusion time *Δ*, and $\rho(r_0)$ is the spin density. This probability $P(r_0|r, \Delta)$ to find a spin in position *r* is dependent on the investigated system. In the case of free diffusion in bulk it has a Gaussian form

$$P(r_0|r, \Delta) = \frac{1}{\sqrt{4\pi D\Delta}} \exp\left(\frac{r^2}{4D\Delta}\right) \tag{S9}$$

which - in combination with **Equation S8** - yields the Stejskal-Tanner equation (**Equation 1**) with the term $(\Delta - \delta/3)$ replaced by $\Delta$.

The probability $P(r_0|r, \Delta)$ to find a molecule in position *r* in the case of free diffusion is depicted in **Figure S17**, varying (**a**) the observation time *Δ* with a fixed value for the diffusion coefficient, which is around the value of *D* for the confined molecules in the porous silicas. **Figure S17** (**b**) shows the probability distribution for different *D* with a fixed value of *Δ* = 6 ms, which corresponds to the value used in the experiments.

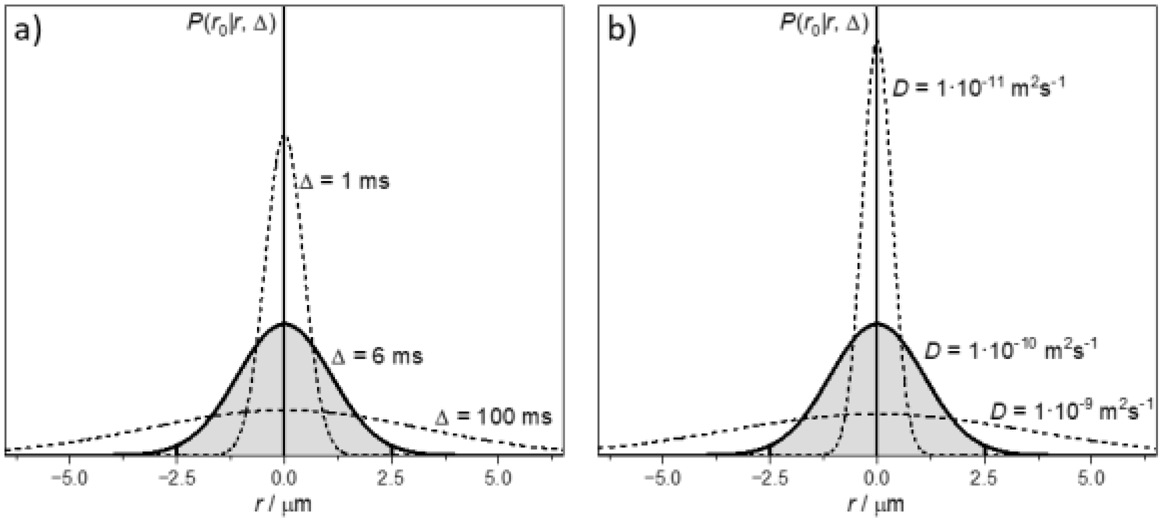


***Figure S17*** *Probability distribution $P(r_0|r, \Delta)$ of finding a molecule starting at position $r_0$ in position r (a) for a variation of observation times Δ with a fixed value for the diffusion coefficient D of 1x10$^{-10}$ m$^2$ s$^{-1}$ and (b) for a variation of D with a fixed value for Δ of 6 ms.*

**Figure S17** shows a broadening of the probability distribution with increasing diffusion time and diffusion coefficient. The silica particles have radii of 2.5 µm or 5.0 µm. This implies that even at the shortest technically possible observation time of $\Delta$ = 6 ms, the probability that a molecule starting in the center of a particle of 2.5 µm radius would diffuse outside the particle would be about 2%. A higher observation time or diffusion coefficient would even increase this effect (for 100 ms: 43%; for 1X10$^{-9}$ m$^2$s$^{-1}$: 47%). In addition, the probability of a molecule to diffuse outside is higher for molecules not starting in the middle of the particle.

However, to avoid an averaging of diffusion in the porous particles with free diffusion in interstitial voids between the particles we have prepared our samples by confining all liquid in the pores, leaving the interstitial space unoccupied, i.e., not filled with liquid. Thus, the diffusing molecules get reflected at the particle interface, modifying the probability distribution $P(r_0|r,\Delta)$. For the case of diffusion in a reflecting sphere, a model has been derived by Balinov et al., where $P(r_0|r,\Delta)$ is described ,[13,14] and **Equation S10** derived [15,16]

$$E(\delta,\Delta,g) = \frac{9[\gamma g\delta R\cos(\gamma g\delta R) - \sin(\gamma g\delta R)]^2}{(\gamma g\delta R)^6}$$
$$+6(\gamma g\delta R)^2 \sum_{n=0}^{\alpha} [j'_n(\gamma g\delta R)]^2 \sum_m \frac{(2n+1)\alpha_{nm}^2}{\alpha_{nm}^2 - n^2 - n} \exp\left(-\frac{\alpha_{nm}^2 D\Delta}{R^2}\right) \frac{1}{[\alpha_{nm}^2 - (\gamma g\delta R)^2]^2} \quad (S10)$$

We employ this model to describe the echo decay curves in **Figure 8**. The reason why this model could not fully describe the whole decay in **Figure 8** (see dashed lines) can be illustrated with **Figure S17b**. The diffusion coefficients extracted for the confined liquids are between 1x10$^{-10}$ m$^2$ s$^{-1}$ and 1x10$^{-11}$ m$^2$s$^{-1}$. In this case the silica particles are still large enough (radii of 2.5 µm or 5.0 µm), such that only a limited share of molecules gets reflected at the particle interface. The effect of the restricted diffusion is therefore not strong enough to describe the entire echo decay. Thus, we attribute most of the deviation from the exponential decay at high $g$ values to the internal magnetic field gradients and only used the initial decay, which is being less affected by the internal gradients, for fitting with the model of diffusion in a closed sphere.

Finally, two fitting approaches were employed and compared, both neglecting the high *g* region due to its shape being dominated by internal gradients. On the one hand we used a biexponential fit, only further considering the fast component; and on the other hand we fitted the model of diffusion in a closed sphere to the initial echo decay at low *g*. The respective values are listed in **Table S4** and **Table S5**, and are summarized in **Figure 11**.

***Table S4.*** *Diffusion coefficients of the molecules confined in silica particles from the model of restricted diffusion in a sphere according to Balinov et al.*[15]

| $D$ / $m^2 s^{-1}$ | phenylethylacetate | phenylethanol | tocopherolacetate | tocopherol |
|---|---|---|---|---|
| Perfect Sil® 300 5 µm | $(9.2 \pm 0.9) \times 10^{-11}$ | $(8.5 \pm 0.8) \times 10^{-11}$ | $(4.4 \pm 0.4) \times 10^{-11}$ | $(3.4 \pm 0.3) \times 10^{-11}$ |
| LiChrospher® Si100 5 µm | $(6.5 \pm 0.6) \times 10^{-11}$ | $(5.9 \pm 0.6) \times 10^{-11}$ | $(3.0 \pm 0.3) \times 10^{-11}$ | $(2.2 \pm 0.2) \times 10^{-11}$ |
| LiChrospher® Si60 5 µm | $(2.8 \pm 0.3) \times 10^{-11}$ | $(2.4 \pm 0.2) \times 10^{-11}$ | $(0.95 \pm 0.09) \times 10^{-11}$ | $(0.80 \pm 0.08) \times 10^{-11}$ |
| LiChrospher® Si100 10 µm | $(11 \pm 1) \times 10^{-11}$ | $(7.1 \pm 0.7) \times 10^{-11}$ | $(4.7 \pm 0.5) \times 10^{-11}$ | $(2.8 \pm 0.3) \times 10^{-11}$ |

***Table S5.*** *Diffusion coefficients of the molecules confined in silica particles from the fast component of a biexponential fit.*

| $D$ / $m^2 s^{-1}$ | phenylethylacetate | phenylethanol | tocopherolacetate | tocopherol |
|---|---|---|---|---|
| Perfect Sil® 300 5 µm | $(9.4 \pm 1.4) \times 10^{-11}$ | $(9 \pm 1) \times 10^{-11}$ | $(4.5 \pm 0.4) \times 10^{-11}$ | $(4.2 \pm 0.5) \times 10^{-11}$ |
| LiChrospher® Si100 5 µm | $(7.1 \pm 0.9) \times 10^{-11}$ | $(5.7 \pm 0.7) \times 10^{-11}$ | $(3.0 \pm 0.4) \times 10^{-11}$ | $(3.6 \pm 0.6) \times 10^{-11}$ |
| LiChrospher® Si60 5 µm | $(4.5 \pm 0.6) \times 10^{-11}$ | $(3.4 \pm 0.5) \times 10^{-11}$ | $(1.4 \pm 0.2) \times 10^{-11}$ | $(1.0 \pm 0.1) \times 10^{-11}$ |
| LiChrospher® Si100 10 µm | $(8.0 \pm 0.9) \times 10^{-11}$ | $(7 \pm 1) \times 10^{-11}$ | $(4.2 \pm 0.4) \times 10^{-11}$ | $(3.6 \pm 0.5) \times 10^{-11}$ |

# Calculation of $\Phi_{WP}$ and corresponding data

## Calculation of $r_a$ (observed reaction rate per catalyst mass)

For batch catalysis:

$$r_a = \frac{(100-conversion)c_aV_a}{t_{reaction}m_{cat}},^{17} \quad (S11)$$

Where $c_a$ is the concentration of the respective alcohol (PE or TP), $V_a$ is the Volume of the reaction solution, $t_{reaction}$ is the reaction time, and $m_{cat}$ is the mass of catalyst (inclusive silica).[18,19]

***Table S6.*** *Reaction conditions for the evaluation of the $\Phi_{WP}$ value for PE:*

| | LiChrospher® Si60 5 µm | LiChrospher® Si100 5 µm | Perfect Sil® 300 5 µm |
|---|---|---|---|
| Conversion / % | 40.33±0.02 | 69.07±0.01 | 46.17±0.02 |
| $t$ / s | 900.0±4.5 | 900.0±4.5 | 900.0±4.5 |
| Catalyst loading / mmol $g^{-1}$ | 0.26±0.03 | 0.26±0.03 | 0.18±0.03 |
| $m_{cat}$ / mg | 23.2±0.1 | 23.1±0.1 | 33.3±0.1 |
| $c_a$ / mol $L^{-1}$ | 0.3000±0.0009 | 0.3000±0.0009 | 0.3000±0.0009 |
| $V_a$ / mL | 2.000±0.006 | 2.000±0.006 | 2.000±0.006 |
| $r_a$ / mol $s^{-1}$ $g^{-1}$ | (1.72±0.08)x$10^{-5}$ | (0.89±0.04)x$10^{-5}$ | (1.08±0.05)x$10^{-5}$ |

***Table S7.*** *Reaction conditions for the evaluation of the $\Phi_{WP}$ value for TP:*

| | LiChrospher® Si60 5 µm | LiChrospher® Si100 5 µm | Perfect Sil® 300 5 µm |
|---|---|---|---|
| Conversion / % | 15.02±0.03 | 26.41±0.02 | 28.49±0.02 |
| $t$ / s | 900.0±4.5 | 900.0±4.5 | 900.0±4.5 |
| Catalyst loading / mmol $g^{-1}$ | 0.26±0.03 | 0.26±0.03 | 0.18±0.03 |
| $m_{cat}$ / mg | 14.8±0.1 | 10.0±0.1 | 15.1±0.1 |
| $c_a$ / mol $L^{-1}$ | 0.02000±0.00006 | 0.02000±0.00006 | 0.02000±0.00006 |
| $V_a$ / mL | 3.000±0.009 | 3.000±0.009 | 3.000±0.009 |
| $r_a$ / mol $s^{-1}$ $g^{-1}$ | (3.8±0.2)x$10^{-6}$ | (4.9±0.3)x$10^{-6}$ | (3.2±0.2)x$10^{-6}$ |

For flow catalysis

$$r_a = \frac{(100-conversion)c_aV_a}{t_{reaction}m_{cat}}, \quad (S12)$$

$$t_{reaction} = \frac{(V_{interparticular\ void}+V_{pore})m_{cat}}{F}, \quad (S13)$$

In case of the flow catalysis, the $t_{reaction}$ is depending on the residence time of the solution inside the packed-bed reactor and is calculated with **Equation S13**.[1] Here, the $V_{interparticular\ void}$ is determined by MIP measurements, while $V_{pore}$ is based on physisorption measurements of the functionalized materials. The reaction volume $V_a$ was calculated with the respective flow rate $F$ (**Equation S14**).[1]

$$V_a = Ft, \quad (S14)$$

*Table S8.* Calculation of $t_{reaction}$

| | LiChrospher® Si60 5 µm | LiChrospher® Si100 5 µm | Perfect Sil® 300 5 µm |
|---|---|---|---|
| $V_{interparticular\ void}$ / $cm^3\ g^{-1}$ | 1.284±0.006 | 1.284±0.006 | 1.284±0.006 |
| $V_{pore}$ / $cm^3\ g^{-1}$ | 0.500±0.005 | 0.98±0.01 | 0.630±0.006 |
| $m_{cat}$ / mg | 450.0±0.1 | 450.0±0.1 | 450.0±0.1 |
| $F$ / mL $min^{-1}$ | 1.000±0.005 | 1.000±0.005 | 1.000±0.005 |
| $t_{reaction}$ / s | 48.000±0.007 | 61.00±0.01 | 52.000±0.008 |

*Table S9.* Reaction conditions for the evaluation of the $\Phi_{WP}$ value for PE at a flowrate of 1 mL $min^{-1}$:

| | LiChrospher® Si60 5 µm | LiChrospher® Si100 5 µm | Perfect Sil® 300 5 µm |
|---|---|---|---|
| Conversion / % | 23.58±0.02 | 43.20±0.02 | 25.20±0.02 |
| $F$ / mL $min^{-1}$ | 1.000±0.005 | 1.000±0.005 | 1.000±0.005 |
| $t_{reaction}$ / s | 48.000±0.007 | 61.00±0.01 | 52.000±0.008 |
| Catalyst loading / mmol $g^{-1}$ | 0.26±0.03 | 0.26±0.03 | 0.18±0.03 |
| $m_{cat}$ / mg | 450±0.1 | 450±0.1 | 450±0.1 |
| $c_a$ / mol $L^{-1}$ | 0.3000±0.0009 | 0.3000±0.0009 | 0.3000±0.0009 |
| $V_a$ / mL | 5.00±0.02 | 5.00±0.02 | 5.00±0.02 |
| $r_a$ / mol $s^{-1}\ g^{-1}$ | $(5.3±0.2)x10^{-5}$ | $(3.1±0.1)x10^{-5}$ | $(4.8±0.2)x10^{-5}$ |

*Table S10.* Reaction conditions for TP at a flowrate of 1 mL $min^{-1}$.

| | LiChrospher® Si60 5 µm | LiChrospher® Si100 5 µm | Perfect Sil® 300 5 µm |
|---|---|---|---|
| Conversion / % | 9.85±0.03 | 21.67±0.02 | 22.25±0.02 |
| $F$ / mL $min^{-1}$ | 1.000±0.005 | 1.000±0.005 | 1.000±0.005 |
| $t_{reaction}$ / s | 48.000±0.007 | 61.00±0.01 | 52.000±0.008 |
| Catalyst loading / mmol $g^{-1}$ | 0.26±0.03 | 0.26±0.03 | 0.18±0.03 |
| $m_{cat}$ / mg | 450±0.1 | 450±0.1 | 450±0.1 |
| $c_a$ / mol $L^{-1}$ | 0.02000±0.00006 | 0.02000±0.00006 | 0.02000±0.00006 |
| $V_a$ / mL | 5.00±0.02 | 5.00±0.02 | 5.00±0.02 |
| $r_a$ / mol $s^{-1}\ g^{-1}$ | $(4.2±0.2)x10^{-6}$ | $(2.9±0.1)x10^{-6}$ | $(3.3±0.1)x10^{-6}$ |

## Calculation of $\Phi_{WP}$

$$\Phi_{WP} = \frac{r_A R_p^2 \rho_{cat}}{C_{s,A} D_{eff}},^{17} \quad \text{(S15)}$$

*Table S11.* Data used for the calculation of $\Phi_{WP}$.

| | |
|---|---|
| $R_p$ / µm | 2.5 |
| $\rho_{cat}$ / $cm^3\ g^{-1}$ | 2.2 |
| $C_{s,A}$(PE) / mol $L^{-1}$ | 0.3000±0.0009 |
| $C_{s,A}$(TP) / mol $L^{-1}$ | 0.02000±0.00006 |

***Table S12.*** *$\Phi_{WP}$ values for PE batch.*

| | LiChrospher® Si60 5 µm | LiChrospher® Si100 5 µm | Perfect Sil® 300 5 µm |
|---|---|---|---|
| $r_a$ / mol s$^{-1}$ g$^{-1}$ | (1.72±0.08)x10$^{-5}$ | (0.89±0.04)x10$^{-5}$ | (1.08±0.05)x10$^{-5}$ |
| $D_{eff}$(PE) / m$^2$ s$^{-1}$ | (2.4±0.2)x10$^{-11}$ | (5.9±0.6)x10$^{-11}$ | (8.5±0.8)x10$^{-11}$ |
| $\Phi_{WP}$ (PE) | 0.033±0.004 | 0.007±0.001 | 0.0058±0.0008 |

***Table S13.*** *$\Phi_{WP}$ values for TP batch.*

| | LiChrospher® Si60 5 µm | LiChrospher® Si100 5 µm | Perfect Sil® 300 5 µm |
|---|---|---|---|
| $r_a$ / mol s$^{-1}$ g$^{-1}$ | (3.8±0.2)x10$^{-6}$ | (4.9±0.3)x10$^{-6}$ | (3.2±0.2)x10$^{-6}$ |
| $D_{eff}$(TP) / m$^2$ s$^{-1}$ | (0.80±0.08)x10$^{-11}$ | (2.2±0.2)x10$^{-11}$ | (3.4±0.3)x10$^{-11}$ |
| $\Phi_{WP}$ (TP) | 0.33±0.05 | 0.15±0.02 | 0.064±0.009 |

***Table S14.*** *$\Phi_{WP}$ values for PE flow at a flowrate of 1 mL min$^{-1}$.*

| | LiChrospher® Si60 5 µm | LiChrospher® Si100 5 µm | | Perfect Sil® 300 5 µm |
|---|---|---|---|---|
| $r_a$ / mol s$^{-1}$ g$^{-1}$ | (5.3±0.2)x10$^{-5}$ | (3.1±0.1)x10$^{-5}$ | | (4.8±0.2)x10$^{-5}$ |
| $D_{eff}$(PE) / m$^2$ s$^{-1}$ | (2.4±0.2)x10$^{-11}$ | (5.9±0.6)x10$^{-11}$ | | (8.5±0.8)x10$^{-11}$ |
| $\Phi_{WP}$ (PE) | 0.10±0.01 | 0.024±0.003 | | 0.026±0.003 |

***Table S15.*** *$\Phi_{WP}$ values for TP flow at a flowrate of 1 mL min$^{-1}$.*

| | LiChrospher® Si60 5 µm | LiChrospher® Si100 5 µm | Perfect Si®l 300 5 µm |
|---|---|---|---|
| $r_a$ / mol s$^{-1}$ g$^{-1}$ | (4.2±0.2)x10$^{-6}$ | (2.9±0.1)x10$^{-6}$ | (3.3±0.1)x10$^{-6}$ |
| $D_{eff}$(TP) / m$^2$ s$^{-1}$ | (0.80±0.08)x10$^{-11}$ | (2.2±0.2)x10$^{-11}$ | (3.4±0.3)x10$^{-11}$ |
| $\Phi_{WP}$ (TP) | 0.36±0.05 | 0.09±0.01 | 0.068±0.009 |

## Complete $\Phi_{WP}$ values for batch and flow catalysis

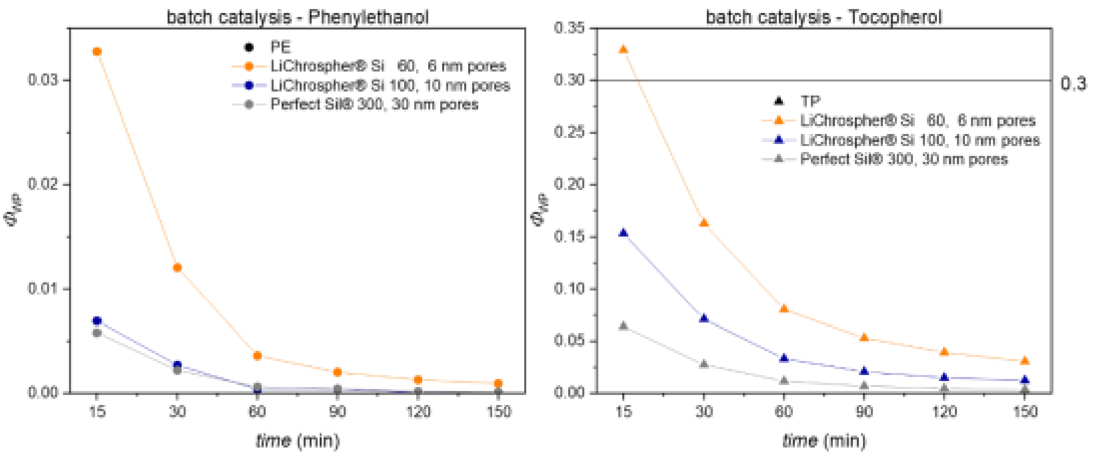


***Figure S18**. All $\Phi_{WP}$ values obtained for batch catalysis.*

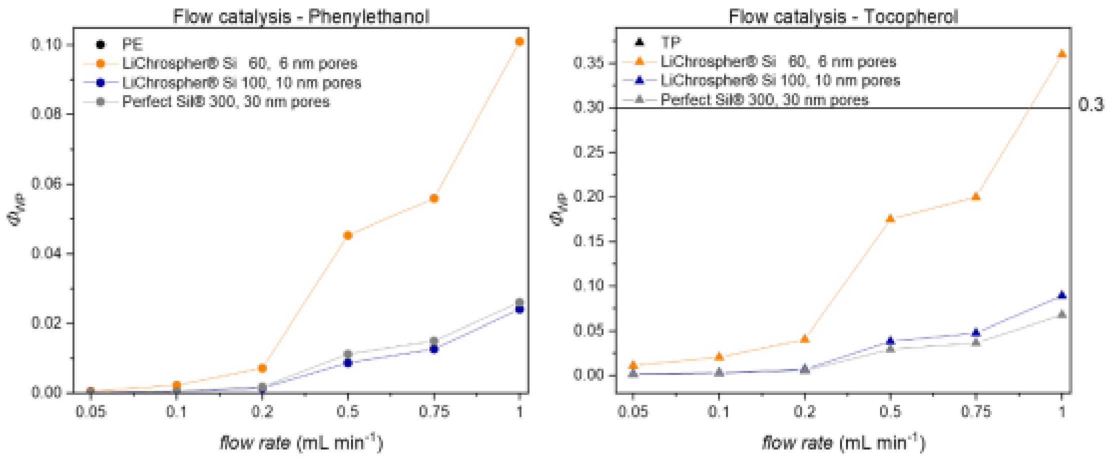


***Figure S19***. *All $\Phi_{WP}$ values obtained for flow catalysis.*

# References


(1) Schulze, J. S.; Brand, R. D.; Hering, J. G. C.; Riegger, L. M.; Schreiner, P. R.; Smarsly, B. M. DMAP Immobilized on Porous Silica Particles and Monoliths for the Esterification of Phenylethanol in Continuous Flow. *ChemCatChem* **2022**, *14* (8), e202101845. https://doi.org/10.1002/cctc.202101845.

(2) Schulze, J. S.; Migenda, J.; Becker, M.; Schuler, S. M. M.; Wende, R. C.; Schreiner, P. R.; Smarsly, B. M. TEMPO-Functionalized Mesoporous Silica Particles as Heterogeneous Oxidation Catalysts in Flow. *J. Mater. Chem. A* **2020**, *8* (7), 4107–4117. https://doi.org/10.1039/c9ta12416k.

(3) Schlumberger, C.; Thommes, M. Characterization of Hierarchically Ordered Porous Materials by Physisorption and Mercury Porosimetry—A Tutorial Review. *Adv. Mater. Interfaces* **2021**, *8* (4). https://doi.org/10.1002/admi.202002181.

(4) Thommes, M.; Smarsly, B.; Groenewolt, M.; Ravikovitch, P. I.; Neimark, A. V. Adsorption Hysteresis of Nitrogen and Argon in Pore Networks and Characterization of Novel Micro- and Mesoporous Silicas. *Langmuir* **2006**, *22* (2), 756–764. https://doi.org/10.1021/la051686h.

(5) Callaghan, P. T. *Translational Dynamics and Magnetic Resonance*; Oxford University Press, 2011. https://doi.org/10.1093/acprof:oso/9780199556984.001.0001.

(6) Weber, D.; Sederman, A. J.; Mantle, M. D.; Mitchell, J.; Gladden, L. F. Surface Diffusion in Porous Catalysts. *Phys. Chem. Chem. Phys.* **2010**, *12* (11), 2619–2624. https://doi.org/10.1039/b921210h.

(7) Elwinger, F.; Pourmand, P.; Furó, I. Diffusive Transport in Pores. Tortuosity and Molecular Interaction with the Pore Wall. *J. Phys. Chem. C* **2017**, *121* (25), 13757–13764. https://doi.org/10.1021/acs.jpcc.7b03885.

(8) Zheng, G.; Price, W. S. Suppression of Background Gradients in (B0 Gradient-Based) NMR Diffusion Experiments. *Concepts Magn. Reson. Part A* **2007**, *30* (5), 261–277. https://doi.org/10.1002/cmr.a.20092.

(9) Washburn, K. E.; Eccles, C. D.; Callaghan, P. T. The Dependence on Magnetic Field Strength of Correlated Internal Gradient Relaxation Time Distributions in Heterogeneous Materials. *J.*

*Magn. Reson.* **2008**, *194* (1), 33–40. https://doi.org/10.1016/j.jmr.2008.05.025.

(10) Hürlimann, M. D. *Effective Gradients in Porous Media Due to Susceptibility Differences*; 1998; Vol. 131.

(11) Kärger, J.; Lenzner, J.; Pfeiffer, H.; Schwabe, H.; Heyer, W.; Janowski, F.; Wolf, F.; Ždanov, S. P. NMR Study of Adsorbate Self-Diffusion in Porous Glasses. *J. Am. Ceram. Soc.* **1983**, *66* (1), 69–72. https://doi.org/10.1111/j.1151-2916.1983.tb09971.x.

(12) Kärger, J.; Pfeifer, H.; Rudtsch, S. The Influence of Internal Magnetic Field Gradients on NMR Self-Diffusion Measurements of Molecules Adsorbed on Microporous Crystallites. *J. Magn. Reson.* **1989**, *85*, 381–387.

(13) Veeman, W. S. Diffusion in a Closed Sphere. *Annu. Reports NMR Spectrosc.* **2003**, *50*, 201–216. https://doi.org/10.1016/S0066-4103(03)50005-9.

(14) Neuman, C. H. Spin Echo of Spins Diffusing in a Bounded Medium. *J. Chem. Phys.* **1974**, 4508–4511. https://doi.org/10.1063/1.1680931.

(15) Balinov, B.; Jönsson, B.; Linse, P.; Söderman, O. The NMR Self-Diffusion Method Applied to Restricted Diffusion. Simulation of Echo Attenuation from Molecules in Spheres and between Planes. *J. Magn. Reson. Ser. A* **1993**, *104* (1), 17–25. https://doi.org/https://doi.org/10.1006/jmra.1993.1184.

(16) Linse, P.; Söderman, O. The Validity of the Short-Gradient-Pulse Approximation in NMR Studies of Restricted Diffusion. Simulations of Molecules Diffusing between Planes, in Cylinders and Spheres. *J. Magn. Reson. Ser. A* **1995**, *116* (1), 77–86. https://doi.org/https://doi.org/10.1006/jmra.1995.1192.

(17) Fogler, H. S. *Elements of Chemical Reaction Engineering*, Sixth.; Pearson, 2022.

(18) Vannice, M. A. *Kinetics of Catalytic Reactions*; Springer Science & Business Media: New York, 2005. https://doi.org/10.1007/b136380.

(19) Mukherjee, S.; Vannice, M. A. Solvent Effects in Liquid-Phase Reactions. I. Activity and Selectivity during Citral Hydrogenation on $Pt/SiO_2$ and Evaluation of Mass Transfer Effects. *J. Catal.* **2006**, *243* (1), 108–130. https://doi.org/10.1016/j.jcat.2006.06.021.
